# In Flight Performance of the MAGIC Magnetoresistive Magnetometer on the RadCube CubeSat

**DOI:** 10.1007/s11214-025-01170-w

**Published:** 2025-05-14

**Authors:** J. P. Eastwood, P. Brown, T. Oddy, M. O. Archer, R. Baughen, I. Belo Ferreira, C. Cobo Torres, E. Cupido, H. Eshbaugh, C. Palla, A. Vitkova, C. L. Waters, B. Whiteside, B. Zabori, A. Hirn, D. Nolbert, D. Milánkovich, Z. G. Kovács, G. Santin, R. Walker

**Affiliations:** 1 Department of Physics, Imperial College London, London, UK; 2 HUN-REN Centre for Energy Research, EK, Budapest, Hungary; 3 Astronika, Warsaw, Poland; 4 C3S LLC, Budapest, Hungary; 5 Directorate of Technology, Engineering and Quality, ESA ESTEC, Noordwijk, The Netherlands; 6 Present Address: CENTRA, Instituto Superior Técnico, Universidade de Lisboa, Lisbon, Portugal; 7 Present Address: AOPP, Clarendon Laboratory, University of Oxford, Oxford, UK; 8 Present Address: MDA Space, Didcot, UK; 9 Present Address: Jet Propulsion Laboratory/California Institute of Technology, Pasadena, CA USA; 10 Present Address: MSSL, University College London, Holmbury St. Mary, UK

**Keywords:** RadCube, Magnetometer, Anisotropic magnetoresistive sensing, CubeSat, Space weather, Geomagnetic observations

## Abstract

In studying space physics, planetary science, and space weather, space-based in situ measurements of the magnetic field are fundamental to understanding underlying physical processes, as well as providing context for other observations. Whilst in many cases instrument design is not severely constrained by the available resource envelope, there are many applications, particularly when using new generations of spacecraft platforms such as CubeSats, that require very low resource sensors. In this context we review the design, development, construction, and flight of the highly miniaturised MAGIC (MAGnetometer from Imperial College) instrument on the RadCube Technology Demonstration CubeSat. MAGIC consists of a boom-mounted (outboard) Anisotropic Magneto-Resistive (AMR) vector sensor connected by harness to a single electronics card inside RadCube. A second inboard AMR vector sensor is mounted on the electronics card. RadCube launched on 17 August 2021 to a sun-synchronous low-Earth polar orbit, with the main mission lasting until April 2022. Routine operations were subsequently extended to the end of 2022, with further special operations in 2023 and 2024 before re-entry on 20 August 2024. Here we review RadCube observations made over more than two years in orbit. Key results from MAGIC on RadCube include meeting ESA space weather magnetic field measurement requirements with both the outboard and inboard sensor, as well as detection of field aligned current signatures at high latitude.

## Introduction

In situ measurement of the magnetic field is crucial across the fields of space physics, space weather and planetary science (Acuña 2002; Balogh 2010; Bennett et al. 2021). In space physics, the magnetic field is fundamental to the properties and behavior of the plasma, and in planetary science magnetic field observations reveal important information about both internal processes and interactions with the space environment. In the field of space weather, interplanetary magnetic field measurements contribute to understanding the geoeffectiveness of solar wind drivers, and magnetospheric magnetic field observations characterise the state of the system and the onset and evolution of geomagnetic storms and substorms (e.g., Eastwood et al. 2017).

In the case of the Earth, magnetic field measurements have been made by a wide variety of satellite missions, particularly in Low Earth Orbit (LEO) (Knipp et al. 2014). For some missions and applications, it is necessary to measure the magnetic field with absolute accuracy and high precision; these form the basis of magnetic field models and deliver key insights into the physical processes by which the Earth’s magnetic field is generated (Olsen and Stolle 2012, 2016). In other investigations, it is sufficient to measure the variation of the magnetic field relative to a model field, as the physical processes of interest are generally related to the structure and dynamics of ionospheric and magnetospheric current systems (Cowley 2000; Milan et al. 2017). For applications relating to space weather monitoring and forecasting, observation of the magnetosphere will ultimately require constellations of satellites and sensors (Morley 2020). This constellation is likely to be heterogenous: for example, the European Space Agency (ESA) Distributed SWE Sensor System (D3S) envisages a constellation of dedicated Small Satellites and CubeSats together with hosted payloads in Low, Medium and Geostationary Earth Orbit (LEO/MEO/GEO) that will ultimately provide the necessary measurements for space weather forecasting services (Kraft et al. 2019).

In general, scientific and operational objectives set measurement requirements that inform the instrument and platform design and guide the necessary resource envelope (e.g., mass, power, data-rate, platform magnetic noise, platform pointing accuracy). However, space applications are often resource limited, and so there is wide interest in developing a new generation of miniaturised instrumentation that can be deployed where resources are insufficient for traditional instrument solutions (Spence et al. 2022). To meet these goals, recent efforts in space magnetometry have focussed on both the development of miniaturised fluxgate sensors (Miles et al. 2016), for example flown on the ELFIN CubeSat (Angelopoulos et al. 2020) and Ex-Alta 1 (Mann et al. 2020), but also the use of anisotropic magnetoresistive (AMR) sensing technology to build a solid-state magnetometer such as implemented on DICE (Fish et al. 2014).

The continuing miniaturisation of flux-gate sensors is highly attractive as they are well-understood and widely used in space applications, with flight heritage demonstrating very stable instrument performance over long periods of time (Forslund et al. 2008). However, their miniaturisation is ultimately limited by the physical nature of the sensing element itself; in particular, sensor noise increases significantly with reduced core diameter (Ripka 2003). Alternatively, the use of magnetoresistive material as a sensing element opens up the possibility of a dramatic reduction in sensor size, but potentially at the expense of more limited performance and stability (e.g. Lenz and Edelstein 2006).

MAGIC (MAGnetometer from Imperial College) is an AMR based magnetometer (Brown et al. 2012, 2014). The primary goal of MAGIC is to offer an order-of-magnitude reduction in resource envelope compared to traditional fluxgate instruments, whilst returning performance commensurate with space weather and space science mission requirements. MAGIC consists of an external vector sensor connected by harness to a single Printed Circuit Board (PCB) electronics card. The PCB also includes MR sensors to provide an inboard vector measurement.

The external sensor consists of a triad of orthogonally mounted single-axis chip sensors, each sensitive to the magnetic field in a single direction. A chip sensor typically consists of four magnetoresistive elements arranged in a Wheatstone bridge configuration as described in Brown et al. (2012). Each of these elements is manufactured as a 2D structure with a magnetized ‘easy’ axis and an orthogonal ‘hard’ sensing direction that is sensitive to the magnetic field. The bridge is imbalanced in the presence a field along the sense direction, and so the bridge voltage provides a measurement of the magnetic field strength on the sensing axis. Whilst many types of MR sensors are available (e.g. Giant MR, Tunnelling MR, etc.), so-called Anisotropic Magneto-Resistance (AMR) is found to have the best detectivity for DC field measurements up to 10 Hz (Stutzke et al. 2005).

The MAGIC design optimises the performance of each chip sensor in two ways (Brown et al. 2012). The first is by using the ‘offset strap’ coil to operate the sensor in a null field environment through closed loop feedback. The second optimisation is to apply a high-frequency bipolar set-reset pulse on a second coil that continuously realigns the sensor magnetisation parallel or antiparallel to the ‘easy’ axis. This constant resetting, or flipping, of the sensor element magnetisation ensures that it operates as optimally as possible and minimises sensor drift. Furthermore, the flipping causes a square-wave output in the bridge voltage, which can be used to interrogate and account for the sensor bridge offset (caused by the fact the four elements of the bridge are not perfectly balanced) during the demodulation process.

The MAGIC heritage includes flight on the CINEMA CubeSat (Archer et al. 2015), and technical development in the context of the Sunjammer solar sail project (Eastwood et al. 2015). Further flight experience has been gained by integration of AMR technology into the SOSMAG instrument (a hosted magnetometer payload on Geo-Kompsat 2A in GEO), where these sensors are used in combination with a miniature fluxgate sensor (Magnes et al. 2020).

**Fig. 1:**
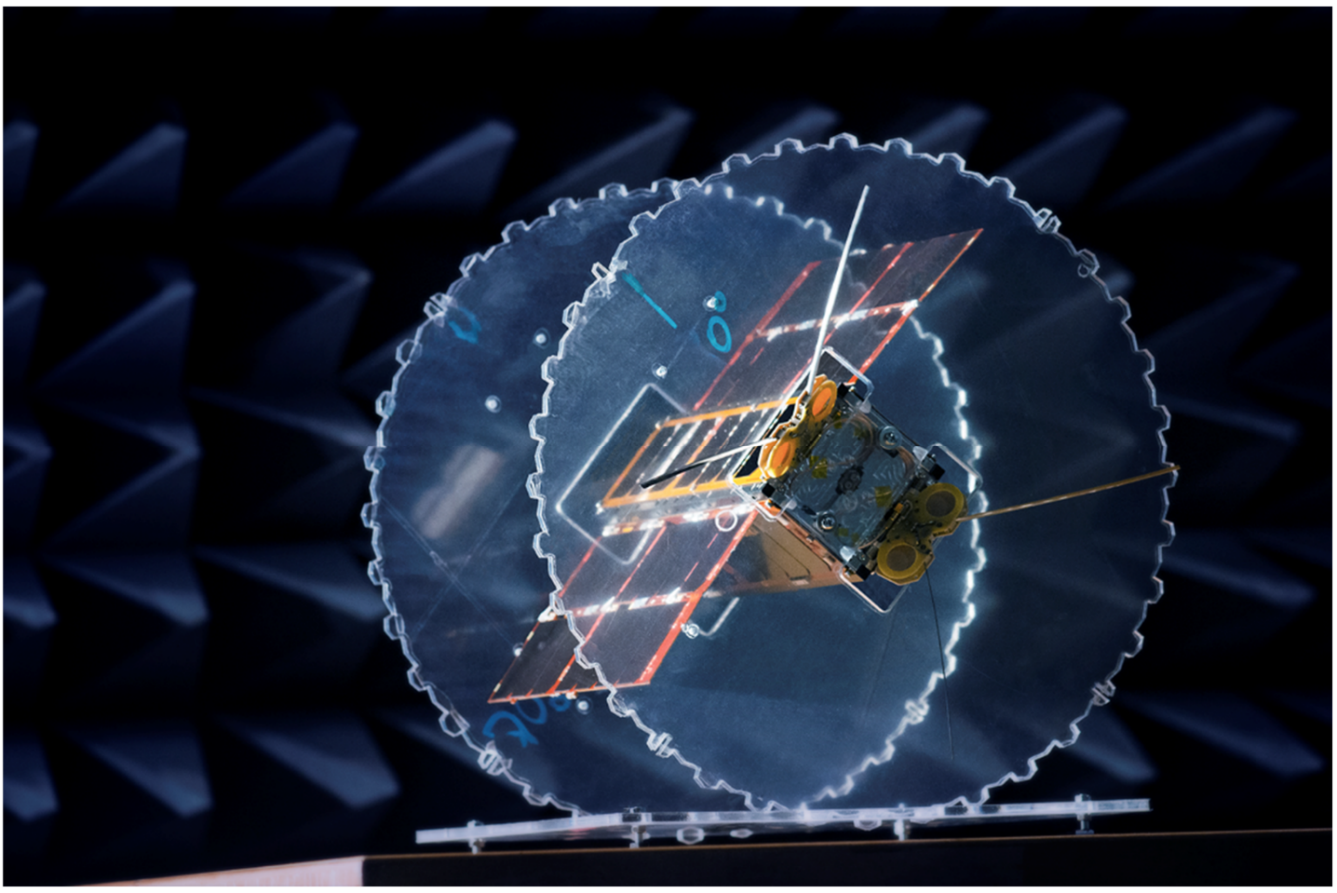
Photograph of the RadCube Proto-Flight Model (PFM) taken during testing. The solar panels and radio antennae are deployed, but the magnetometer boom is not deployed

In this review we present the most recent implementation of MAGIC on the RadCube CubeSat (Fig. 1) which launched in 2021. The objective of the RadCube project is to demonstrate in flight, through successful mission operation, the future use and applicability of the RadCube system for space weather research (and nowcast/forecast) services and general radiation damage monitoring for commercial electronic components. The project, conducted via the ESA General Technology Support Programme and managed by the ESA CubeSat Systems Unit, covers the demonstration of both the novel 3-U CubeSat platform developed by Complex Systems and Small Satellites (C3S LLC), Hungary, and the RadMag energetic particle and magnetic field science payload (Zabori et al. 2018). The CubeSat is designed to be 3-axis stabilized, and the Attitude Determination and Control System (ADCS) consists of both magnetotorquers and reaction wheels. The RadMag payload occupies a 1.2 U payload volume and consists of the RadTel energetic particle detector comprising two orthogonally mounted detectors (HUN-RED EK, Hungary, consortium lead), the MAGIC instrument (Imperial College London, UK), a novel tape-spring boom system (Astronika, Poland) and two CHIMERA radiation hardness assurance test boards (ESA). RadMag is thus designed to provide an integrated space weather monitoring capability in an extremely limited resource envelope.

The review is organised as follows. Details of the MAGIC instrument requirements, design, manufacture, and testing are presented in Sect. 2. We then discuss the in-flight performance, beginning in Sect. 3 with a summary of the mission timeline as relevant to the MAGIC instrument. The calibration and performance of the outboard boom-mounted sensor is presented and discussed in Sect. 4, and the inboard sensor is similarly presented in Sect. 5. A summary is provided in Sect. 6.

## MAGIC Instrument

### Requirements

The primary objective of MAGIC on RadCube is to demonstrate improved sensor performance in LEO relevant for space weather monitoring applications. The mission-level requirement was set as achieving a 5% accuracy in measurement of the magnetic field, with a goal to achieve 1% accuracy and reach the ESA MR-015-M space weather product specification as described in the Product Specification Document issued by the ESA Space Safety Programme (ESA 2013). It is important to note that this should be interpreted as the level of error achieved following in-flight calibration. This led to the definition of a more specific set of requirements for the RadCube magnetic field measurements which are summarised in Table 1.

**Table 1:** MAGIC instrument requirements

Parameter	Requirement
Directionality	3-axis
Range [nT]	+/ − 60,000
Orthogonality error [°]	≤0.1
Measurement absolute accuracy^*^	<5%
Noise limit [pTrms/$\sqrt{\text{Hz}}$]	≤500
Temperature coefficient [nT / °C]	≤+/ − 1
Sampling rate [vectors/s]	1.0 / 10

^*^taking into account all possible error sources

The first three requirements (directionality, range and orthogonality) were placed to ensure the instrument can measure the magnetic field at the altitude of RadCube in any orientation without saturation. The next three requirements were placed based on accuracy requirements. The measurement absolute accuracy from all possible error sources applies to the field magnitude and flows from the mission requirement. These include the performance of the MAGIC sensor (including e.g. sensor orthogonality error), the deployed boom position accuracy (discussed further below), the magnetic noise generated by the satellite, and the satellite orientation knowledge and accuracy. The ADCS system was required to be able to detumble RadCube and maintain Absolute Performance and Knowledge Errors of 5° and 0.2°, respectively. The noise performance requirements are consistent with typical AMR performance (Brown et al. 2014; Magnes and Diaz-Michelena 2009; Stutzke et al. 2005). The temperature coefficient requirement reflects the fact that the zero-field offsets of AMR sensors are known to be highly sensitive to temperature, which may complicate the calibration of the instrument. Finally, the nominal sampling rate was set at 1 vector/s, sufficient to capture magnetic field structure associated with field aligned currents. A requirement for a higher cadence sampling rate at 10 vectors/s was also included, for use in non-routine observations (e.g. boom deployment). For completeness, we note that there were other project requirements and these are mentioned where relevant in the remainder of Sect. 2.

### Instrument Overview

**Fig. 2:**
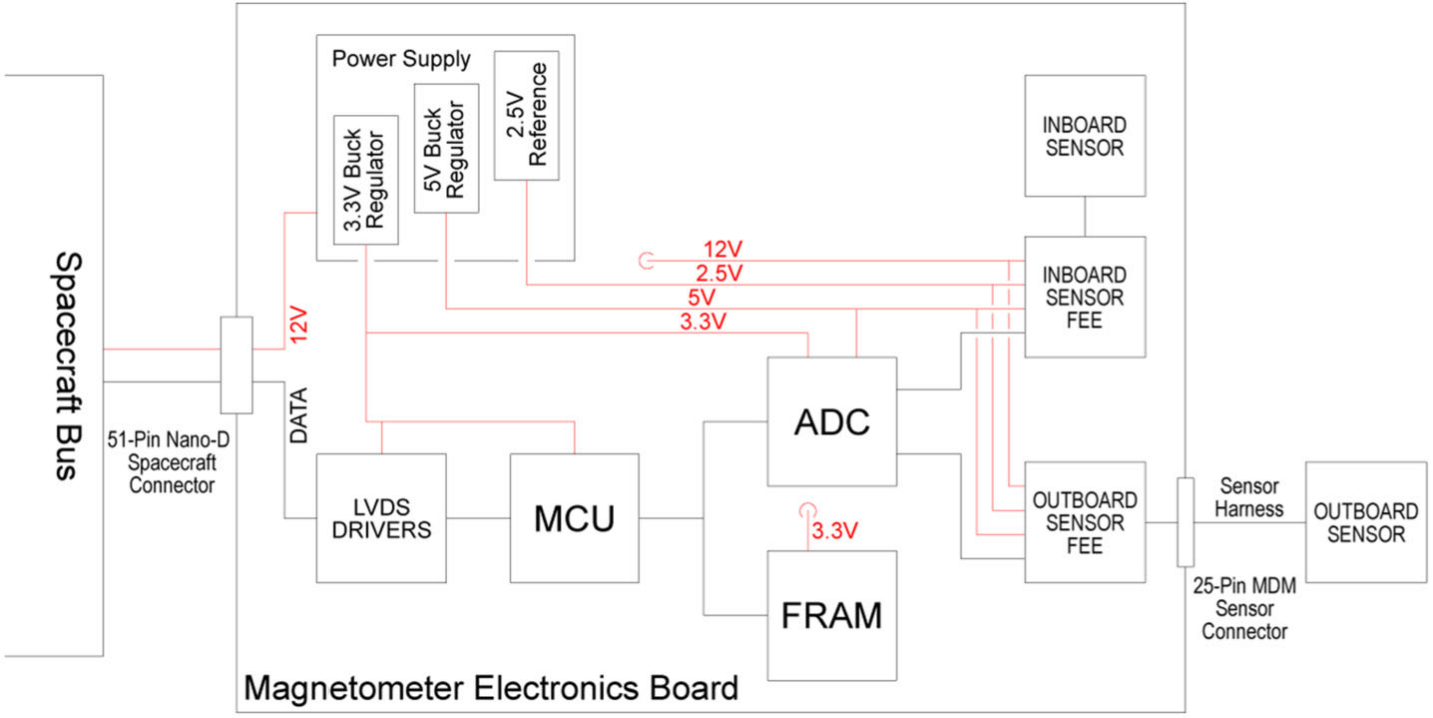
Block diagram of RadCube MAGIC. Data connections are shown in black, and power connections are shown in red

The MAGIC instrument on RadCube is derived from the instrument flown on the CINEMA CubeSat. The basic principle of operation is described by Brown et al. (2014) and a block diagram is shown in Fig. 2. For RadCube, the design again consisted of a single three-axis outboard (OB) sensor connected by harness to a single PCB, with a further three-axis inboard (IB) sensor located on the PCB. The OB sensor is thus designed to be boom mounted, and to act as the primary sensor. The IB sensor characterizes platform magnetic interference and provides redundancy in the event of OB sensor failure. Each sensor is connected to its own dedicated Front End Electronics (FEE) circuit, which then connects via an Analogue-Digital Converter (ADC) to the Magnetometer Control Unit (MCU). Instrument configurations are stored in Ferro-electric Random Access Memory (FRAM), and Low Voltage Differential Signaling (LVDS) drivers provide a digital data interface to the spacecraft bus. The instrument requires a single 12 V power rail from the spacecraft bus and the power supply circuit delivers analogue and digital voltage lines as required, described in more detail below.

The front-end analogue magnetometer closed control loop was largely unchanged from CINEMA. However, several changes were introduced to improve the performance and versatility of the instrument. These include:

1. Changing the AMR sensors from the Honeywell HMC1001 used on CINEMA to the HMC1021Z. The HMC1021Z has the same package as the sensors used in the CINEMA design but its dynamic range (±6 gauss) is better optimised for measurements in LEO compared to the previously used HMC1001. The HMC1021Z also requires lower power for full feedback operation.
2. A reduction in the volume of the OB sensor to approximately half that flown on CINEMA.
3. The amplitude of the set-reset current for the HMC1021Z is lower than that of the HMC1001 (0.5 A for 2 μs versus 3 A). This allowed for the elimination of the 17 V rail that was used in the CINEMA design (Brown et al. 2014).
4. The set-reset circuit on the hybrid sensor was simplified by replacing 4 individual dies with a single H-bridge MIC 4422 inverting MOSFET driver. The CINEMA set/reset pulses were generated by a square wave used to drive an H-Bridge with a capacitor in series with the set-reset straps. In the RadCube design each side of the H-Bridge is explicitly driven with a pulse from a microcontroller.
5. Addition of intelligence through use of an Atmel AVR ATmega128 microprocessor. This facilitates standard communications protocols, with more flexible instrument management and commanding (e.g. data rates), providing capabilities that were not available with the CINEMA implementation.
6. The RadCube design runs off a single 12 V rail (as opposed to the 4 regulated rails on CINEMA). The 12 V is used as the bias for generation of the set-reset current spikes while the analogue and digital circuit rails (5 V and 3.3 V) are generated from the 12 V using two buck regulators.

**Fig. 3:**
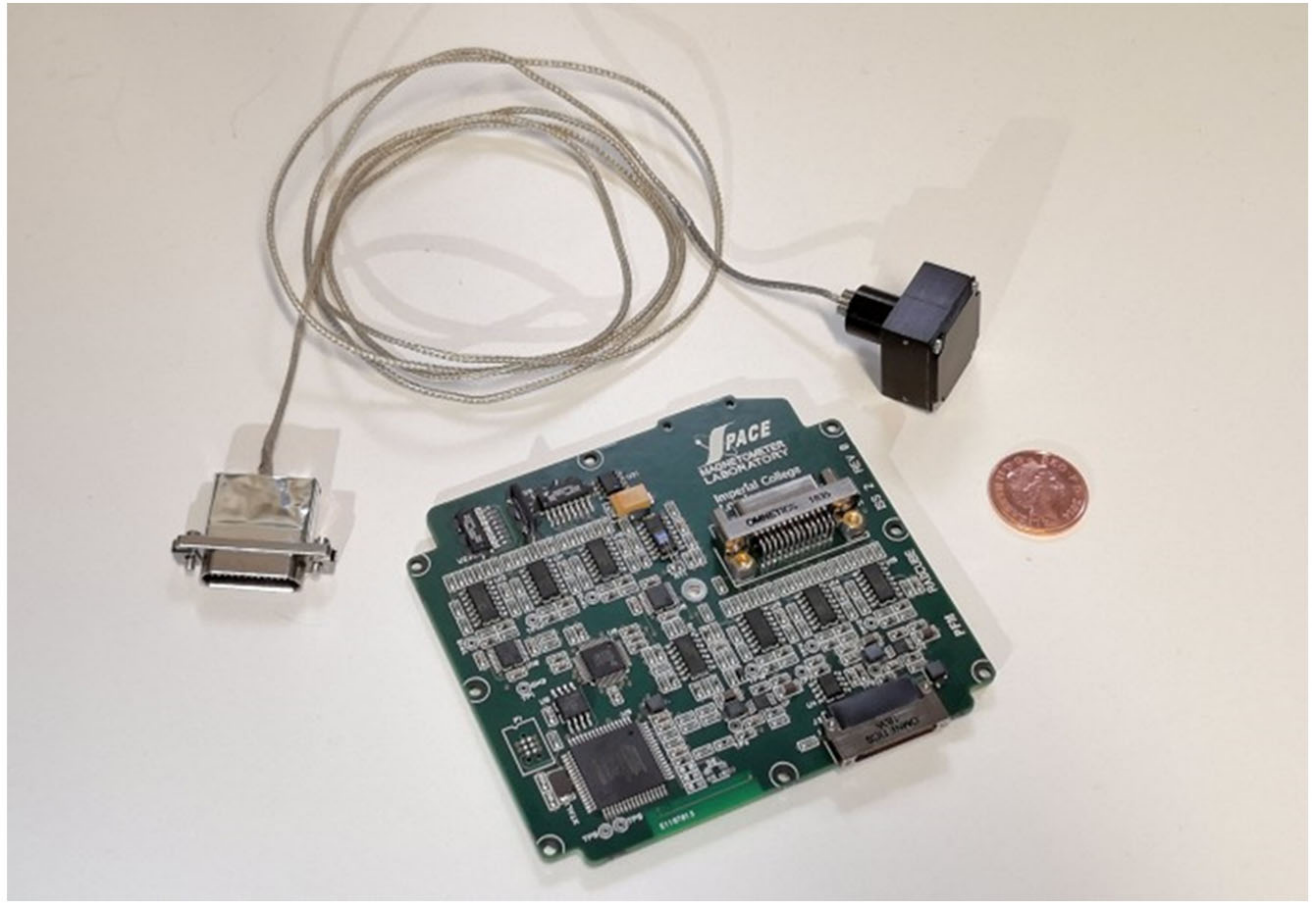
Photograph of the RadCube MAGIC Proto-Flight Model

To illustrate the subsequent sections, Fig. 3 shows a photograph of the MAGIC Proto-Flight Model which launched on RadCube, consisting of the electronics card (containing the Inboard sensor), and the Outboard sensor and harness. A 1 pence GBP coin (diameter 20.3 mm) is included for scale.

### Mechanical Design

#### OB Sensor Mechanical Design

The most visible change to the OB sensor design relative to CINEMA is a reduction in the height of the sensor enclosure to 12.6 mm. This was feasible because there was significant unused volume in the final flight version of CINEMA, as design margin implemented at the start of the CINEMA mission was not required in the end. The second major change was to accommodate the tape-spring boom interface. On CINEMA, the OB sensor was mounted to a plate at the end of a stacer boom. This meant that the base of the OB sensor mechanical chassis had a flat mounting design and was attached by screws. On RadCube, the OB sensor mechanical chassis was redesigned such that an asymmetric cylindrical hollow tube protrudes from the bottom of the chassis (visible in Fig. 3). The tape-spring boom is attached by screws to this cylindrical structure by means of an interface plate, both made of titanium. Within the sensor chassis, the OB sensor hybrid tile also required modification to accommodate the routing of the harness.

#### Boom and OB Sensor Harness

**Fig. 4:**
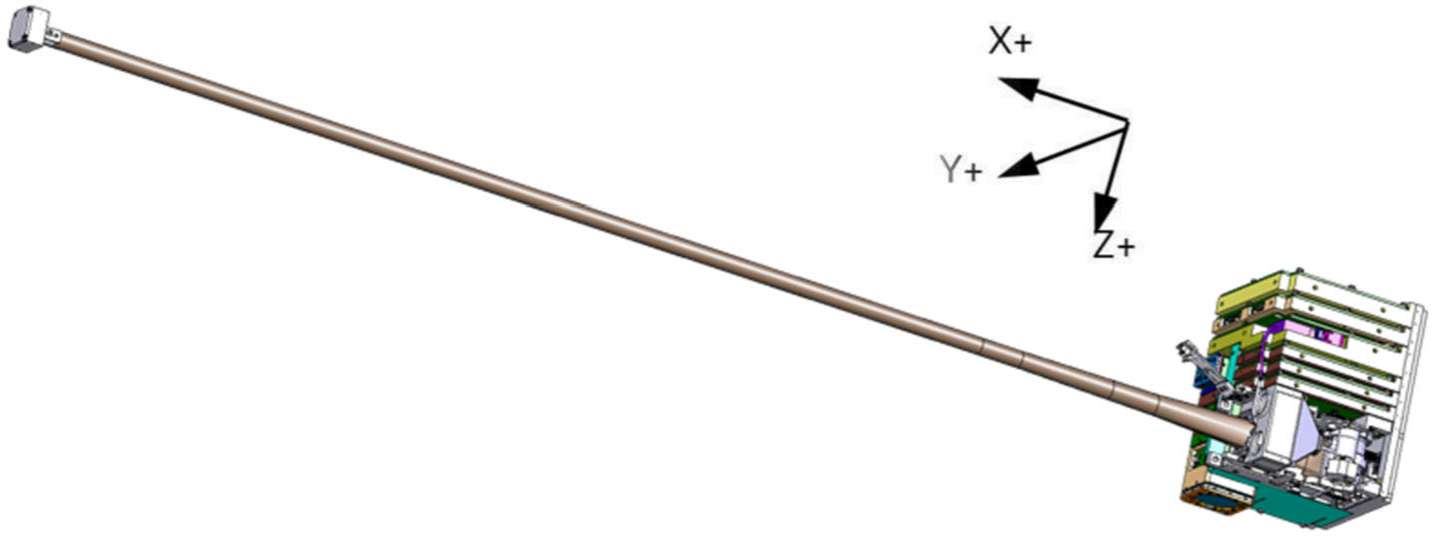
Illustration of the RadMag payload with magnetometer boom deployed. The MAGIC outboard sensor is visible at the end of the boom. The platform coordinate system is also shown, with the x-direction aligned to the boom. The boom deploys perpendicular to the plane containing the solar panels in the anti-sunward direction

As noted in Sect. 1, RadCube provides a technology demonstration of a very highly miniaturised deployable tape-spring boom to minimize the spacecraft magnetic noise environment at the OB sensor. Tape-spring booms have a long space flight heritage, for example being used as antennas for radio experiments (Grygorczuk et al. 2023), but are not typically used as magnetometer booms. A key technology demonstration goal of RadCube was to explore the use of such a boom for magnetometer measurements, because it offers an extremely compact resource envelope. In particular, the boom deployed angular accuracy was required to be ±1.5°/m which was confirmed in testing in laboratory environment prior to launch. As with all tape-spring booms, the boom tape is wound on a reel. A DC motor rotates the bobbin, and as the boom tape deploys, it relaxes into a cylindrical form. Figure 4 shows an illustration of the RadMag payload (size 10 cm × 10 cm × 12 cm), with the boom fully deployed to a length of 80 cm.

On CINEMA, the harness was wrapped around the stacer boom, but this was not possible with the tape-spring system. The design solution on RadCube was to route the harness inside the tape spring boom, i.e. within the hollow cylinder formed by the boom. Before deployment, the sensor was clamped to the spacecraft body, with the clamp released prior to boom deployment by a thermal knife.

The harness connecting the OB sensor to the MAGIC electronics card consists of a core of 20 copper (36 AWG) polyester-insulated wires with a topcoat of amide-imide, applied twice. The wires are arranged into 4 strands, each containing 5 wires, and are twisted with a RHRL (Right-Hand Regular-Lay) configuration and a twist of 10 mm. The bundle is covered by Aracon braid, except for the last 100 mm which is coated with another, non-magnetic braid. The minimum bending radius of this cable is 7 mm, and this enabled it to be stowed within a small volume within the boom system. The harness diameter is approximately 1.6 mm, with mass of approximately 8.5 g/m. As on CINEMA, an MDM 25-way connector was used to connect the harness to the electronics card. Although this connector is relatively large, it ensures a robust electrical termination on the PCB of the wires used in the extremely low mass OB sensor harness.

#### Electronics Card

The magnetometer electronics is housed on a single 86.5 mm by 95 mm card, populated on both sides and consistent with internal RadMag layout and volume envelope constraints. In addition to the Micro-D connector used to attach the harness, a 51-pin Nano-D connector is used to connect the Magnetometer card to the RadMag payload backplane bus.

### Thermal Design

The thermal environment experienced by MAGIC in LEO was expected to be similar to that experienced by CINEMA. The qualification and acceptance temperature ranges for the electronics card were defined as [−30 °C, +70 °C] and [−20 °C, +60 °C], respectively. Prior to RadCube, the electronics card was qualified in the range [−50 °C, +60 °C], but based on the component selection it is however capable of operating up to +80 °C.

Regarding the OB sensor, the qualification and acceptance temperature ranges were [−70 °C, +70 °C] and [−60 °C, +60 °C], respectively. As the sensor design had previously been qualified in the range [−80 °C, +80 °C], no heater was required in the RadCube design. However, the AMR sensor gain is sensitive to temperature and so to passively control the thermal environment of the OB sensor, the exterior of the mechanical housing was treated with a black anodised coating. We also note that the potting of the sensor improves the thermal stability of the sensor environment. A non-magnetic thermistor was co-located with the AMR sensor triad on the hybrid tile, with MAGIC returning this data to the ground to be used as part of the calibration procedure.

### Electrical Design

The electrical design used for the implementation of MAGIC on RadCube draws extensively from CINEMA flight experience but includes two new principal developments. The first is the inclusion of intelligence via the addition of a microprocessor (the Atmel AVR ATmega128). The processor controls the sampling of the on-board ADC (on CINEMA this function was performed by the main bus); as on CINEMA, the implementation on RadCube uses an 8-channel 24-bit ADC. In practice, this provides 114 pT digital resolution. The processor also generates the stream of set-reset pulses and their duration, with a flipping frequency of 512 Hz. The digital interface to the bus is implemented using a pair of bi-directional LVDS drivers.

The second development is the addition of a buck converter for voltage conditioning. As illustrated in Fig. 2, the main bus rail is 12 V, coming from the spacecraft. The main rail is further regulated on the electronic card to generate 5 V (for analogue rails and reference voltage) and 3.3 V for the microprocessor.

### Software Design

The introduction of instrument intelligence represents a significant enhancement in the capabilities of the MAGIC instrument. The software is implemented as an interrupt driven loop written in C, with some small elements in assembler, and includes the following key functions:

1. A buffered, interrupt-controlled full-duplex serial line between the instrument and the spacecraft bus.
2. Packet encoding and decoding using Serial Line Internet Protocol (SLIP), and Cyclic Redundance Check (CRC) validation of packets;
3. Interrupt-driven clock, providing millisecond-precision instrument time from boot (to a maximum value of 49.7 days past boot).
4. Programmable pulse and demodulation-reference generation for the analogue front-end electronics.
5. On-die and external ADC control and sampling, for the acquisition of magnetic field science data, and housekeeping data such as sensor temperatures and bus voltages.

The software is split into boot and application modules. At power-on, following memory checks the instrument switches into a selectable version of the application software. In boot mode, an application may be loaded automatically after a configurable delay ranging between 100 ms and approximately two hours, with switch-over preemptable at any time to allow instrument patching or configuration changes. Alternatively, this application switch-over can be commanded manually by the spacecraft. An onboard watchdog can be enabled to provide additional tolerance in the event of unexpected processor behaviour.

Interrupts are used to identify the reception of data, and to ensure the outgoing interface is supplied with a continuous stream of data. Telemetry packets are assembled in buffers, adding other protocol elements before transmission. Data sampling from the Central Processing Unit (CPU)’s internal 10-bit ADC (used for sampling of HK voltage and temperatures) and the external 24-bit ADC (used to sample the magnetic field data from the inboard and outboard sensors) is controlled based upon the instrument time and buffered prior to being assembled into the outgoing science and housekeeping data packets. Internal CPU counter/timers are used to generate an internal time reference, and to provide hardware clocking for the analogue front end.

MAGIC has two primary cadences but can be commanded into a variety of different modes, as described in Table 2. The packet structure is fixed, but the rate at which it is sent to the spacecraft varies according to the operating mode. Science packets are produced at a rate of one-tenth the sampling cadence of the primary sensor. Housekeeping packets, containing critical parameters such as bus and secondary voltages, sensor temperature, etc., are generated at one tenth the rate of science packets. MAGIC primarily operated in NORMAL mode in-flight with occasional transitions to FAST or FASTGRAD mode on special occasions such as boom deployment.

**Table 2:** MAGIC operating modes. Note that either the OB or IB sensor can be designated as primary

Name	Data Rate Primary (vectors/s)	Data Rate Secondary (vectors/s)
NORMAL	1	0.1
FAST	10	1
GRAD	1	1
FASTGRAD	10	10
SLOW	0.1	0.01
BURST	25	2.5

### Manufacture and Testing

The RadCube project model philosophy entailed construction of an Engineering Qualification Model (EQM) followed by a Proto-Flight Model (PFM). Qualification testing was performed using the EQM, and acceptance testing was performed on the PFM. The construction, test, and delivery of the PFM in mid-late 2020 was heavily impacted by the Covid-19 pandemic. Starting in March 2020, activities in the UK were highly restricted. Engineering staff required special permission to work on site, and the restrictions in working practice (e.g. social distancing and limits on lab occupancy) meant that many activities could not proceed as normal. For example, nationally imposed restrictions meant it was not possible to thermally test the PFM electronics card due to the necessary facilities not being available for use. Limited thermal testing of the PFM sensor recovered offset temperature coefficients of x: 0.92 nT/°C, y: 0.62 nT/°C, z: 1.85 nT/°C. It was also not possible to verify the orthogonality requirement of the OB sensor (Table 1), again due to facility non-availability. The impact of international travel restrictions was also a severe obstacle to efficient assembly and system-level testing. These impacts further reduced the overall period of unit level performance testing. Nevertheless, despite this very severe stress-test of normal working conditions, the MAGIC instrument was successfully delivered on schedule at the end of 2020.

Performance testing of the PFM sensor was carried out at Imperial College London, with the sensor being placed in a jig inside a triple-layer mu-metal can, connected to the PFM electronics on the bench. A coil system in the can allows known magnetic fields to be applied along the axis of the can. The jig allows the sensor to be placed in different orientations, and so enables testing of sensor performance in three orthogonal directions, depending on how the sensor is oriented relative to the can axis. A variety of test signals were applied including impulse, square wave, chirps (high/low freq), and linear sweep. Each test was repeated with three different sensor orientations. These tests satisfactorily demonstrated the linearity of the instrument and the ability to measure the required field strengths.

A second test to establish the noise performance of the instrument was performed in an overnight test in null field, inside the mu-metal can. Measurements were made at 10 vectors/s and the Fast Fourier Transform (FFT) of this data was used to calculate the Power Spectral Density (PSD) at 1 Hz. The noise levels on each axis were found to be $\mathrm{B}_{\mathrm{x}} = 430\text{ pT}/\sqrt{\text{Hz}}$, $\mathrm{B}_{\mathrm{y}} = 79\text{ pT}/\sqrt{\text{Hz}}$, $\mathrm{B}_{\mathrm{z}} = 292\text{ pT}/\sqrt{\text{Hz}}$. The sensor axes are nominally aligned with the spacecraft coordinate system illustrated in Fig. 4, with the +X direction pointing along the boom away from RadCube. The final properties of MAGIC are listed in Table 3.

**Table 3:** RadCube MAGIC Properties

Physical Dimension	Electronic card: 95 mm × 87 mm × 10 mm
	OB sensor: 21 mm × 21 mm × 12 mm
Mass	OB sensor and harness: 23 g
	Electronics card including IB sensor: 38 g
Power	0.48 W
Range	±63,000 nT per axis
Digital Resolution	114 pT
Sensor Noise	<500 pT/$\sqrt{\text{Hz}}$ at 1 Hz, per axis
Measurement Cadence	0.1 vector/s – 25vector/s

## RadCube MAGIC in-Flight Timeline

Before discussing the in-flight performance in detail, we first review the overall RadCube MAGIC in-flight timeline, so as to place subsequent results in context. After full integration, the entire RadCube CubeSat was degaussed at the ESTEC Ulysses coil facility to minimise its magnetic moment. RadCube was modelled as composed of one dipole and two quadrupoles, with 22 degrees of freedom. The total dipolar magnetic moment was reduced from 74.95 mAm^2^ (5.6% total rms of residuals - goodness of fit) to 6.22 mAm^2^ (14.2% total rms of residuals - goodness of fit). RadCube was then integrated as an auxiliary payload on Vega Flight 19 and successfully launched from Kourou Spaceport at 01:47 am (UTC) on 17 Aug 2021 (10:47 pm 16 Aug 2021 local time), to a Sun-synchronous orbit at 551 km altitude and 97.56° inclination. Table 4 summarises important MAGIC-related events and milestones during the mission.

**Table 4:** RadCube MAGIC timeline

Date	Event
2021 Aug 17	Launch Vega Flight 19 (VV19)
2021 Oct 05	Health Check
2021 Oct 28	MAGIC boom deployment
2021 Oct 29	Health Check
2021 Nov 10	Start of nominal MAGIC observations
2022 Feb 08	End of Outboard sensor functionality
2022 Apr 04	Inboard sensor set as primary sensor from 20:00 UT
2022 May 04	Start of extended mission
2022 Dec 24	End of extended mission
2023 Nov 24-28	Health check interval
2024 Aug 20	Re-entry

The first data from MAGIC was returned on 5 Oct 2021. Whilst the OB sensor was not deployed at this time, the short ‘health check’ procedure confirmed the functionality of the instrument, returning 15 minutes of data consisting of 5 minutes each of NORMAL, FAST, and GRAD mode data. This demonstrated the satisfactory operation of MAGIC in flight, with data received at the appropriate cadence, and mode switching as commanded. The boom was deployed on 28 Oct 2021, during which time MAGIC was operational; this data is examined in detail in Sect. 4.4. A second health check identical in form to the first was performed on 29 Oct 2021. The post boom deployment health check showed that the OB sensor performance was improved compared to the IB data, and significantly less affected by spacecraft magnetic fields.

On 10 Nov 2021, nominal operations started with the OB sensor as the primary sensor. The performance of the OB sensor is reviewed in Sect. 4, focussing on four extended intervals of magnetometer data acquired in December 2021. The OB sensor subsequently exhibited degraded performance and functionality and could no longer be used after 8 Feb 2022.

Given the loss of the OB sensor as the nominal science phase progressed, the performance of the IB sensor was explored more carefully. In particular, it was noticed that during certain operational modes, RadCube appeared to be magnetically quiet. From 4 Apr 2022 the IB sensor was set as the primary sensor and its performance is analysed in Sect. 5.

## Outboard (OB) Sensor Performance

In this section we review and discuss the performance of the OB sensor. We first present an analysis of four intervals of extended data acquired in late December 2021. During these intervals, MAGIC was operating in NORMAL mode, with an OB sensor measurement cadence of 1 vector/s. In Sect. 4.1 we first describe the attitude independent calibration procedure and in Sect. 4.2 we summarise the OB calibration parameters and performance.

Although the RadCube ADCS system was able to control the orientation of RadCube relatively precisely, it was found that the ADCS solution reported to ground did not correctly recover RadCube’s orientation. Our approach to quaternion reconstruction is described in Sect. 4.3, which enabled the recovery of RadCube observations in a geophysical reference frame. These results demonstrate that the MAGIC instrument on RadCube met both the mission requirement and goal in terms of observing the magnetic field.

In Sect. 4.4, the boom deployment interval is analysed in detail. We apply the calibration parameters that were calculated in flight to this data, and also examine the OB sensor noise performance pre- and post-deployment.

Finally in Sect. 4.5 we review the overall performance of the OB sensor through the mission, focussing on technical challenges and a discussion of the manner in which the OB sensor is thought to have ultimately failed. This provides important insight for the future development and use of the highly miniaturised technologies demonstrated by RadCube.

### Attitude Independent Calibration Procedure

The calibration procedure applied to the RadCube data is essentially identical to that developed and used by Archer et al. (2015) to analyse MAGIC data from CINEMA.

#### L1 Data

The level 1 magnetic field data, $\boldsymbol{B}_{L1}$ is measured in nT and is converted from the engineering unit data $\boldsymbol{B}_{\mathrm{Eng}}$ according to a known scaling, established on the ground, where

1 $$ \boldsymbol{B}_{L1} \left [ \text{nT} \right ] = \frac{63{,}000}{524{,}288} \boldsymbol{B}_{\mathrm{Eng}} \left [ \mathrm{Eng} \right ]. $$

It is also necessary to check the integrity of time-tags of the 1 s resolution data, as the data pipeline can occasionally introduce a timestep $\Delta t = 0\text{ s}$ or $\Delta t = 2\text{ s}$ which must be corrected.

#### L1a Data (Time Corrected)

The next step in the calibration procedure is to generate L1a data which is time-corrected based on comparing the observed data with a field model. RadCube orbit data is used to compute $\boldsymbol{B}_{\mathrm{IGRF}}$, the expected magnetic field at the location of RadCube based on the IGRF-13 model. First removing anomalous data, defined as $\left \vert \frac{{B}_{L1}}{{B}_{\mathrm{IGRF}}} -1 \right \vert >0.25$, from the calculation, the cross-correlation of $|\boldsymbol{B}_{\mathrm{IGRF}}|$ and $|\boldsymbol{B}_{L1}|$ is used to identify corrected spacecraft time where $t_{\mathrm{corrected}} = t_{\mathrm{RC}} - t_{\mathrm{offset}}$. This is then used to define $\boldsymbol{B}_{ L1 a}$ where

2 $$ \boldsymbol{B}_{L1a} \left ( t_{\mathrm{corrected}} \right ) = \boldsymbol{B}_{L1} \left ( t_{\mathrm{RC}} - t_{\mathrm{offset}} \right ). $$

The time offset $t_{\mathrm{offset}}$ is typically found to be of the order of a few seconds. The anomalous data, as defined above, is then removed from the L1a data.

#### L1b Data (Constant Gains and Offsets Applied)

The L1b data is computed by minimizing the squared difference between $|\mathrm{B}_{ L1 b}|$ and $|\mathrm{B}_{\mathrm{IGRF}}|$ assuming constant gains ${G}_{\mathrm{C}}$ and offsets ${O}_{\mathrm{C}}$ where

3 $$ \boldsymbol{B}_{L1b} = \boldsymbol{G}_{C}^{-1} \left ( \boldsymbol{B}_{L1a} - \boldsymbol{O}_{C} \right ) = \left ( \textstyle\begin{array}{c@{\quad}c@{\quad}c} {G}_{Cx}^{-1} & 0 & 0\\ 0 & {G}_{Cx}^{-1} & 0\\ 0 & 0 & {G}_{Cx}^{-1} \end{array}\displaystyle \right ) \left \{ \left ( \textstyle\begin{array}{c} {B}_{L1a,x}\\ {B}_{L1a,y}\\ {B}_{L1a,z} \end{array}\displaystyle \right ) - \left ( \textstyle\begin{array}{c} {O}_{Cx}\\ {O}_{Cy}\\ {O}_{Cz} \end{array}\displaystyle \right ) \right \}. $$

$\mathrm{G}_{\mathrm{c}}$ and $\mathrm{O}_{\mathrm{c}}$ are thus free parameters derived from the least-squares minimization. This step is performed first because the constant gain and offsets are expected to be the biggest contribution to the calibration process. A temperature-dependent offset correction is subsequently applied, as described in Sect. 4.1.5.

#### L1c Data (Orthogonal Sensor Coordinate System)

Up to this point, the magnetic field data is expressed in a non-orthogonal coordinate system aligned to the sensor axes. L1c data places the magnetic field observations in an orthogonal coordinate system and is computed by minimizing the squared difference between $|{B}_{ L1 c}|$ and $|{B}_{\mathrm{IGRF}}|$ assuming constant orientations of the sensor axes. The angular orientation of the $i$th sensor axis in spherical polar coordinates is $(\theta _{\mathrm{i}}, \varphi _{\mathrm{i}})$ and we expect $(\theta _{\mathrm{x}}, \varphi _{\mathrm{x}}) = (90^{\circ}, 0)$, $(\theta _{\mathrm{y}}, \varphi _{\mathrm{y}}) = (90^{\circ}, 90^{\circ})$, $(\theta _{\mathrm{z}}, \varphi _{\mathrm{z}}) = (0, 0)$. The L1c data is then calculated as

4 $$ \boldsymbol{B}_{L1c} = \boldsymbol{A}^{-1} \boldsymbol{B}_{L1b} = \boldsymbol{A}^{-1} \boldsymbol{G}_{C}^{-1} \left ( \boldsymbol{B}_{L1a} - \boldsymbol{O}_{C} \right ) $$

where

5 $$ \boldsymbol{A} = \left ( \textstyle\begin{array}{c@{\quad}c@{\quad}c} \sin \theta _{x} \cos \phi _{x} & \sin \theta _{x} \sin \phi _{x} & \cos \theta _{x}\\ \sin \theta _{y} \cos \phi _{y} & \sin \theta _{y} \sin \phi _{y} & \cos \theta _{y}\\ \sin \theta _{z} \cos \phi _{z} & \sin \theta _{z} \sin \phi _{z} & \cos \theta _{z} \end{array}\displaystyle \right ). $$

#### L2 Data (Temperature Dependent Offsets)

Finally, L2 data is computed by minimizing the squared difference between $|{B}_{L2}|$ and $|{B}_{\mathrm{IGRF}}|$ assuming constant gains ${G}_{\mathrm{T}}$ and temperature dependent offsets ${O}_{\mathrm{T}}$ where

6 $$ \boldsymbol{B}_{L2} = \boldsymbol{G}_{T}^{-1} \left ( \boldsymbol{B}_{L1c} - \boldsymbol{O}_{T} \right ), $$

7 $$ \boldsymbol{O}_{T} = \boldsymbol{O}_{T,1} T+ \boldsymbol{O}_{T,2}, $$

and the temperature is given in °C. By combining all these steps, it can be shown that

8 $$ \boldsymbol{B}_{L2} = \boldsymbol{G}^{-1} \boldsymbol{A}^{-1} \left ( \boldsymbol{B}_{L1a} - \left ( \boldsymbol{O}_{1} T+ \boldsymbol{O}_{2} \right ) \right ) $$

where

9 $$ \boldsymbol{G}^{-1} = \boldsymbol{G}_{T}^{-1} \boldsymbol{G}_{C}^{-1} $$

10 $$ \boldsymbol{O}_{1} = \boldsymbol{A G}_{C} \boldsymbol{O}_{T,1} $$

11 $$ \boldsymbol{O}_{2} = \boldsymbol{O}_{C} + \boldsymbol{A G}_{C} \boldsymbol{O}_{T,2} $$

### Outboard Sensor Calibration Results

As mentioned in Sect. 3, four extended intervals of largely continuous OB data were acquired in late December 2021. The calibration procedure described in Sect. 4.1 was applied to each of these intervals independently, and the calibration parameters are shown in Table 5. The sensor angular orientations recovered by the calibration process closely agree across all events, and the standard deviations are less than 0.1°, with the exception of the $\theta _{\mathrm{y}}$ component. The gains are also found to be remarkably stable across all four intervals. The offsets are much smaller than the typical ambient field, and the increased uncertainty in their values is related to the fact that in strong field regimes (i.e. where the ambient field strength is much greater than the offset value), the gain and angles are more important than the offsets, and therefore easier to constrain.

**Table 5:** Summary of outboard calibration parameters for four observation intervals

Interval	1	2	3	4	**Average**	Std
Start	25/12/2021 23:00	26/12/2021 20:00	27/12/2021 20:00	29/12/2021 20:00		
End	26/12/2021 09:00	27/12/2021 09:00	28/12/2021 09:00	30/12/2021 09:00		
Time Offset [s]	0	3	10	3	**4**	4.2
*θ*_x_ [°]	88.691	88.797	88.932	88.85	**88.818**	0.101
*ϕ*_x_ [°]	−0.003	−0.003	−0.002	−0.004	**−0.003**	0.001
*θ*_y_ [°]	89.232	88.793	88.744	88.922	**88.923**	0.219
*ϕ*_y_ [°]	91.863	91.945	91.869	91.781	**91.865**	0.067
*θ*_z_ [°]	0.009	0.011	0.01	0.01	**0.01**	0.001
*ϕ*_z_ [°]	0.002	0.003	0.002	0.002	**0.002**	0
L2 O_1,x_ [nT/degC]	−7.2	−5.11	−2.86	−5.48	**−5.16**	1.78
L2 O_1,y_ [nT/degC]	−6.57	−7.14	−6.25	−6.1	**−6.51**	0.46
L2 O_1,z_ [nT/degC]	3.27	0.65	−2.51	1.69	**0.77**	2.44
L2 O_2,x_ [nT]	439.83	350.9	217.48	342.1	**337.58**	91.43
L2 O_2,y_ [nT]	495.74	709.28	739.95	662.59	**651.89**	108.85
L2 O_2,z_ [nT]	−45.38	70.46	49.75	21.01	**23.96**	50.48
L2 G_x_	1.029	1.031	1.03	1.03	**1.03**	0.001
L2 G_y_	1.009	1.011	1.01	1.011	**1.01**	0.001
L2 G_z_	1.014	1.018	1.021	1.018	**1.018**	0.003
L2 |B| RMSE [nT]	121.211	114.593	141.876	128.478	**126.54**	11.691
L2 |B| RMSE Frac. Error	0.004	0.003	0.004	0.004	**0.004**	0

**Fig. 5:**
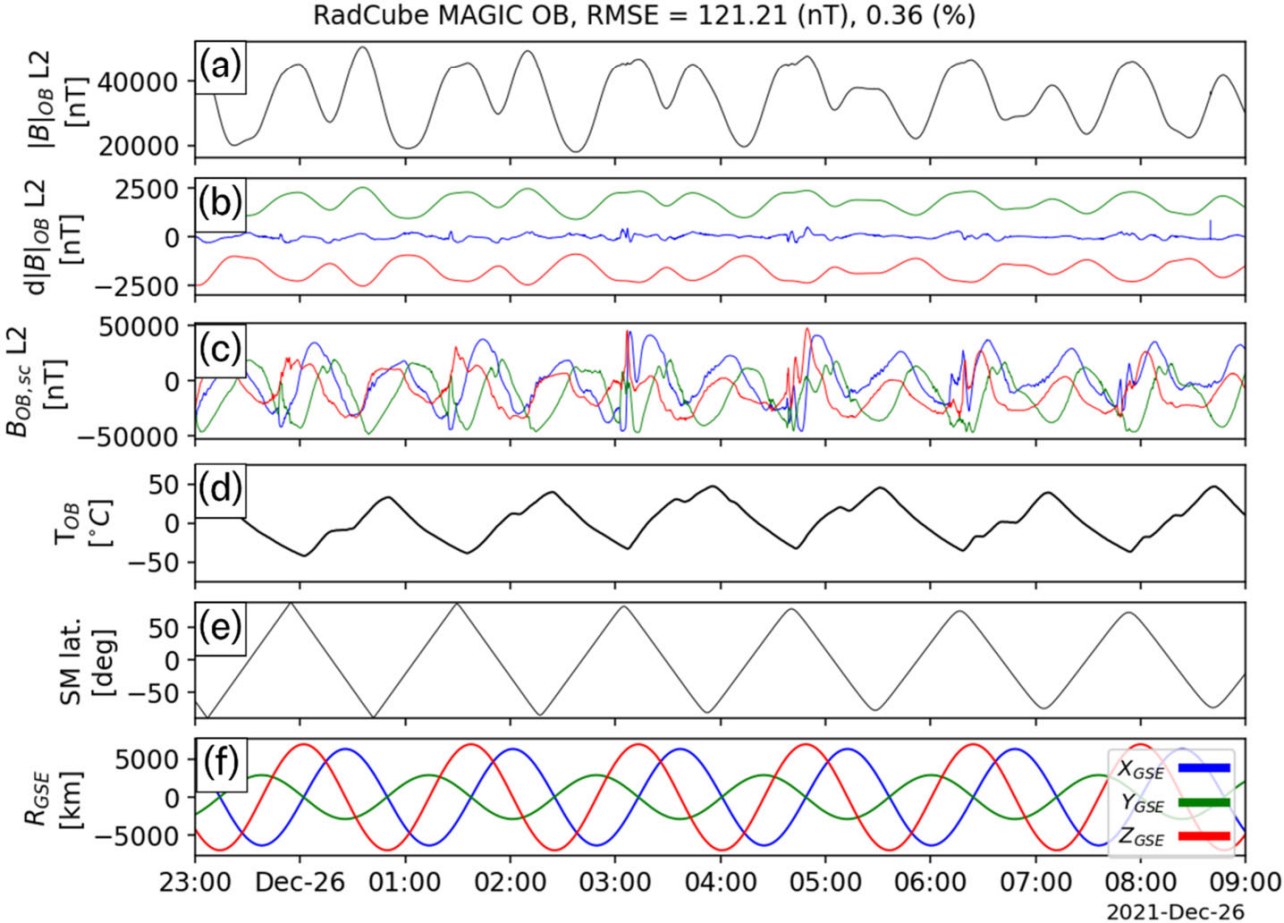
RadCube MAGIC Outboard data – interval 1 2021-12-25 23:00 - 2021-12-26 09:00. Panel (a): L2 calibrated magnetic field strength measured by RadCube. (b) Difference between the observed and model magnetic field strength (blue), with 5% error envelope (red/green). (c) L2 calibrated magnetic field in the sensor coordinate system (x: blue, y: green, z: red). (d) Temperature of the Outboard sensor. (e) Solar Magnetic latitude of RadCube. (f) Location of RadCube in Geomagnetic Solar Ecliptic coordinates (x: blue, y: green, z: red)

**Fig. 6:**
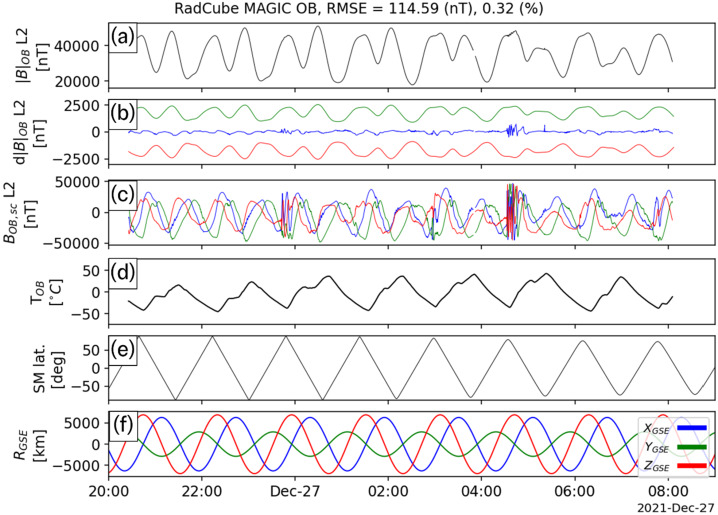
RadCube MAGIC Outboard data – interval 2 2021-12-26 20:00 - 2021-12-27 09:00, in the same format as Fig. 5

**Fig. 7:**
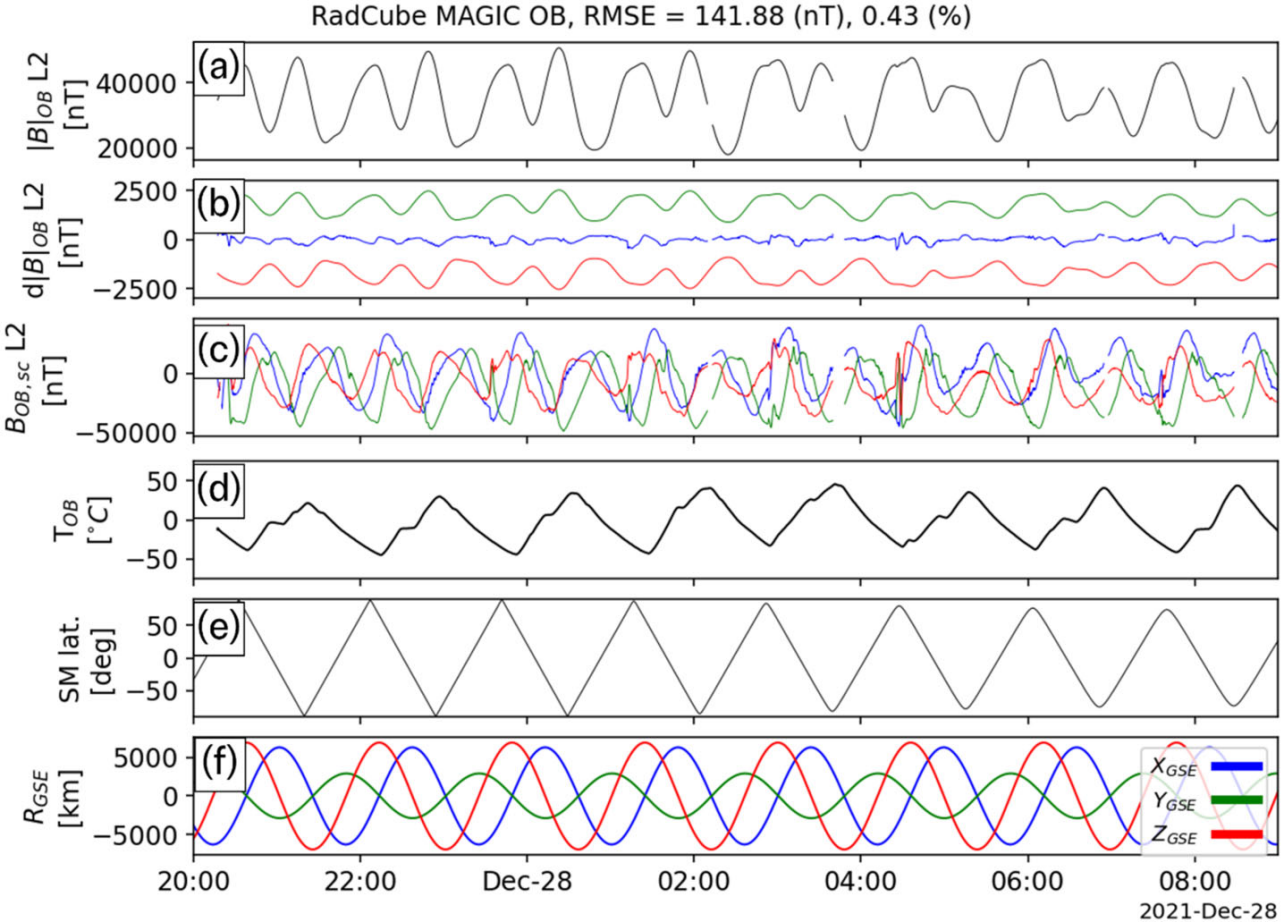
RadCube MAGIC Outboard data – interval 3 2021-12-27 20:00 - 2021-12-28 09:00, in the same format as Fig. 5

**Fig. 8:**
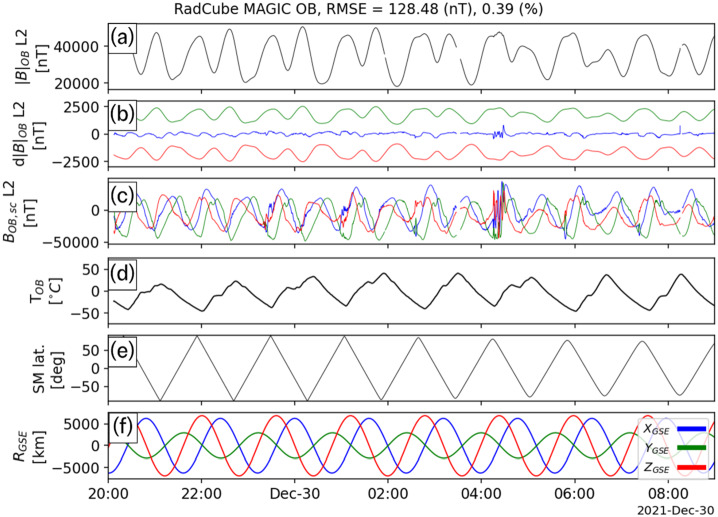
RadCube MAGIC Outboard data – interval 4 2021-12-29 20:00 - 2021-12-30 09:00, in the same format as Fig. 5

Figure 5, Fig. 6, Fig. 7, and Fig. 8 show the L2 calibrated OB magnetic field data for each of the four intervals, respectively. The format of each figure is the same. Panel (a) shows $|{B}_{OB,L2}|$ and in all four cases $|{B}_{OB,L2}|$ smoothly varies, and there is no evidence for steps, spikes or other intermittent deviations. Small gaps in data coverage are caused by removal of observations above the threshold described in Sect. 4.1.2. Panel (b) shows $\text{d}|{B}_{OB}| = |{B}_{OB,L2}| - |{B}_{\mathrm{IGRF}} |$ in blue, with the 5% error envelope shown in green/red. The difference between the measured and expected magnetic field strength is typically of the order of 100 – 200 nT. The difference varies with time, and intervals where it is more pronounced correspond to times when RadCube was changing attitude rapidly. This can be seen in Panel (c) which shows $\boldsymbol{B}_{OB,L2}$ in the orthogonal sensor coordinate system (nominally equivalent to the spacecraft coordinate system). Rapid variations in the components of the field in the sensor frame are due to the change in orientation of RadCube. Panel (d) shows the temperature of the Outboard sensor. The temperature decreases on the nightside and increases on the dayside. It should be noted that during these intervals the sensor is in the shadow of the satellite to control the amount of sensor heating and this is discussed further in Sect. 4.5. Panel (e) shows the latitude of RadCube in Solar Magnetic coordinates; each interval consists of approximately 8 orbits, with the exception of the first interval that covers approximately 6 orbits. Panel (f) shows the position of RadCube in Geocentric Solar Ecliptic coordinates. This is included for context and confirms the orientation of the orbital plane as when x_GSE_ is positive, y_GSE_ is negative. The orbit is such that RadCube descends from northern to southern latitudes on the dayside in the morning sector. To quantify the error in the measured magnetic field strength, we compute the Root Mean Square Error (RMSE) and the fractional RMSE for all four intervals; these are summarised in Table 5. The average RMSE is found to be 126.54 nT, and the fractional RMSE is 0.4%. This represents an improved performance relative to CINEMA where the RMSE was found to be 1.23% (Archer et al. 2015) and well within both the 5% mission requirement and the 1% mission goal.

### Quaternion Reconstruction Applied to OB Data

In the absence of a sufficiently accurate ADCS solution, an alternative approach was used to find quaternions to rotate the data from the sensor coordinate system to a geophysical coordinate system. These were generated based on the MAGIC magnetic field data alone, following the procedure of Archer et al. (2015) and described here for completeness. The starting point is the calibrated L2 data expressed in the orthogonal sensor coordinate (SC) system, i.e. $\mathbf{B}_{\text{L2},\text{SC}}$. At each instant in time, a quaternion exists that rotates this data from the SC coordinate system directly into the Earth Centred Inertial J2000 (ECI-J2000) coordinate system. Two independent vector measurements are required to find this quaternion, for example the expected magnetic field direction, $\mathbf{B}_{\mathrm{IGRF},\text{ECI}}$ (here known) and direction to Sun (here unknown).

The magnetic field data at two points in time is used to constrain the problem and find the best estimate of the quaternion. At time $t_{\mathrm{i}}$, the available information is used to construct the family of possible solutions based on $\mathbf{B}_{\text{L2},\text{SC}}$ ($t = i$) and $\mathbf{B}_{\mathrm{IGRF},\text{ECI}}$ ($t = i$). In essence, this can be thought of as a simple rotation of the observed field in sensor coordinates to along the IGRF direction in ECI-J2000 frame, followed by an arbitrary rotation about the IGRF field. We then select from this family the quaternion $q_{\mathrm{i}}$ that minimises the angle between $\mathbf{B}_{\text{L2}, \text{ECI}}$ ($t= i + 1$) and $\mathbf{B}_{\mathrm{IGRF},\text{ECI}}$ ($t = i + 1$). The reconstruction is effectively Eulerian in time, as $\mathbf{B}_{\text{L2},\text{SC}}$ ($t = i$) and $\mathbf{B}_{\text{L2},\text{SC}}$ ($t = i + 1$) are used to constrain $q_{\mathrm{i}}$.

The reconstruction assumes that the sensor is rotating slowly in the ECI system and that the observed change in $\mathbf{B}_{\text{L2},\text{SC}}$ is due to a changing magnetic field at RadCube, principally due to its orbit, and not due to changes in the orientation of RadCube. To remove any higher-frequency fluctuations in the measurement that may influence the quaternion reconstruction, the data are low-pass filtered, with a cut-off frequency of 0.1 Hz for the OB sensor.

Finally, although the RadCube attitude is relatively stable, there are periods where the orientation of RadCube changes more rapidly, on timescales of 10 s – 100 s. This can be seen for example in Fig. 5 at 04:45 UT. These changes are not sufficiently fast to invalidate the assumptions of the quaternion reconstruction. However, these variations obscure other physical signals such as field aligned currents that exist on similar timescales.

**Fig. 9:**
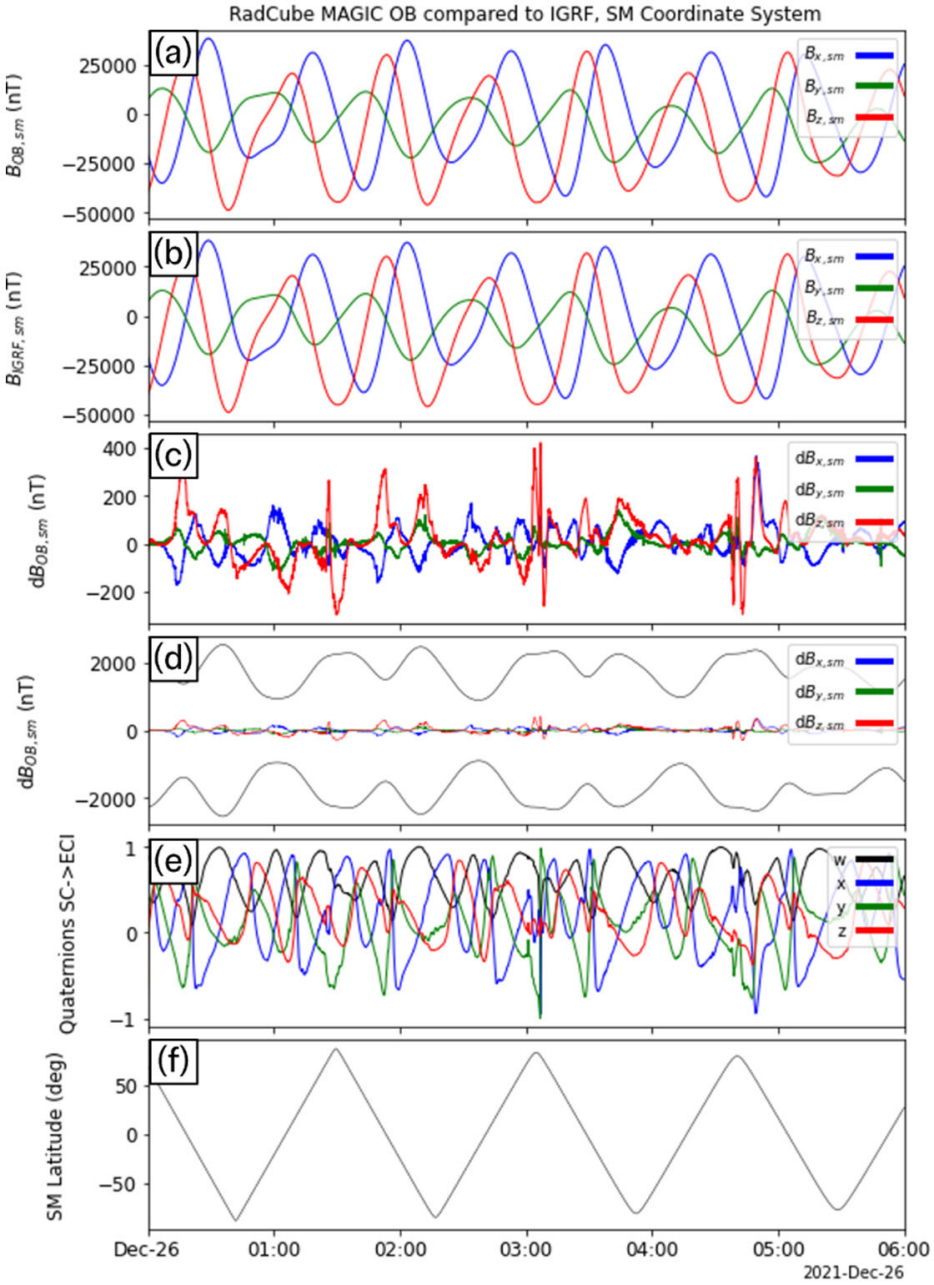
RadCube MAGIC data 2021-12-26 00:00 - 2021-12-26 06:00 in the SM coordinate system and compared to IGRF. Panel (a): L2 calibrated magnetic field data measured by RadCube in the SM coordinate system (x: blue, y: green, z: red). (b) IGRF magnetic field expected at the location of RadCube in SM coordinates (x: blue, y: green, z: red). (c) Difference between each component of the observed and model magnetic field (x: blue, y: green, z: red) (d) The same data as panel (c), but also showing the 5% error envelope based on the magnetic field strength. (e) Reconstructed quaternions (w: black, x: blue, y, green, z: red). (e) Latitude of RadCube in SM coordinates

Having established the quaternion solution, the RadCube L2 magnetic data was then rotated into ECI-J2000, and subsequently into Solar Magnetic coordinates. Figure 9 shows the results of this analysis for the first interval of OB data (corresponding to the interval shown in Fig. 5) between 00:00 UT and 06:00 UT. Panels (a) and (b) show the measured magnetic field and the expected IGRF field, respectively. The data is shown in the SM coordinate system, and very good agreement is observed on this scale. Panels (c) and (d) both show the difference in each component, on two different scales. Panel (c) shows that the difference is typically less than 100-200 nT, but also quite variable. Panel (d) shows the difference in the context of the mission requirements, and clearly demonstrates that the RadCube data are well within the required accuracy. For context panel (e) shows the timeseries of the reconstructed quaternions. Each quaternion component generally varies relatively smoothly validating the method’s assumptions. However, at times, the variation is more rapid, and this also corresponds to periods where the error in the vector magnetic field components increases. Comparing with Fig. 5, these intervals e.g. at just after 03:00 UT, correspond to times when the RadCube orientation was changing rapidly. Finally, panel (f) shows the latitude of RadCube in SM coordinates.

**Fig. 10:**
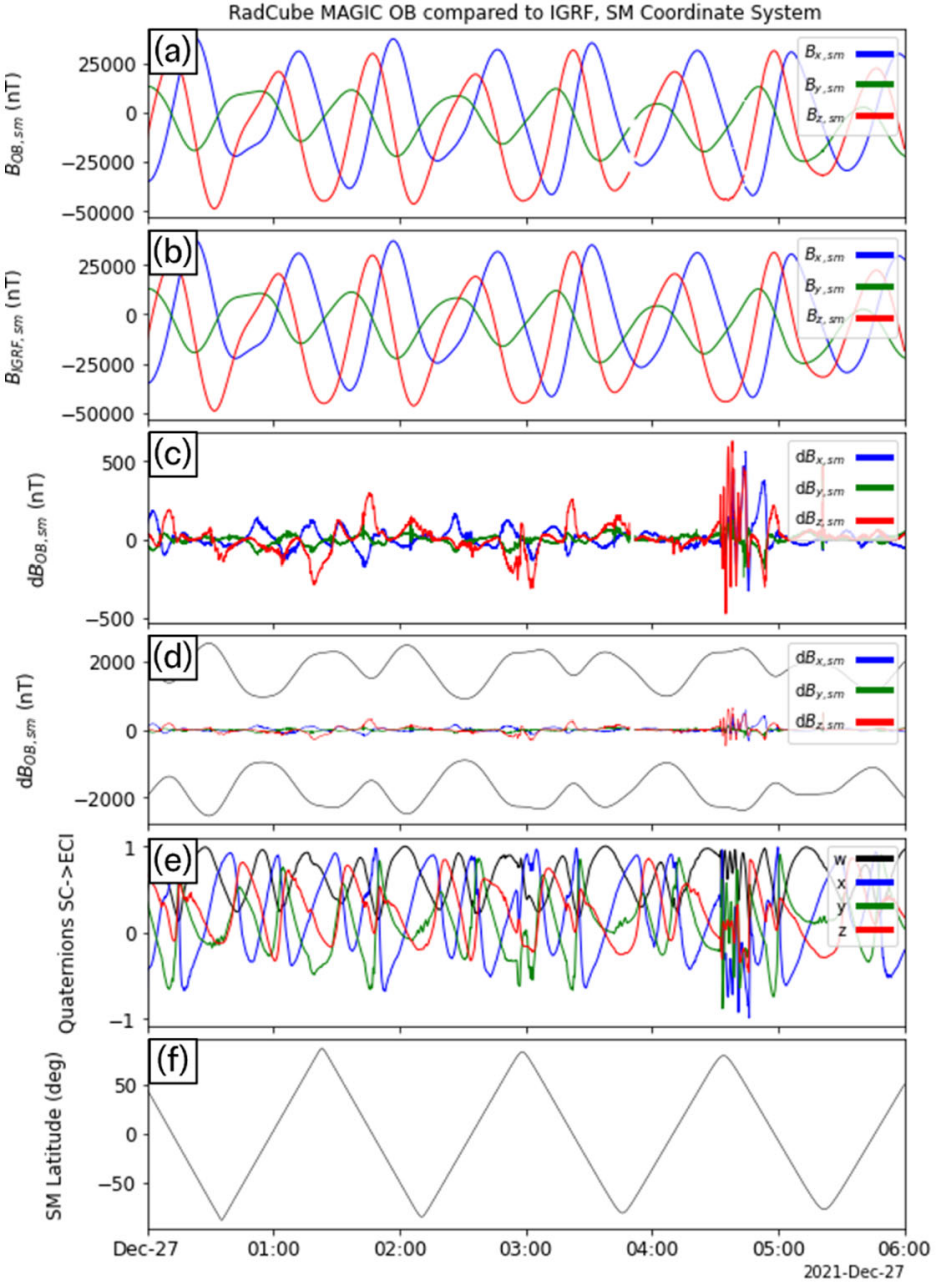
RadCube MAGIC data 2021-12-27 00:00 - 2021-12-27 06:00 in the SM coordinate system and compared to IGRF. Data are shown in the same format as Fig. 9

**Fig. 11:**
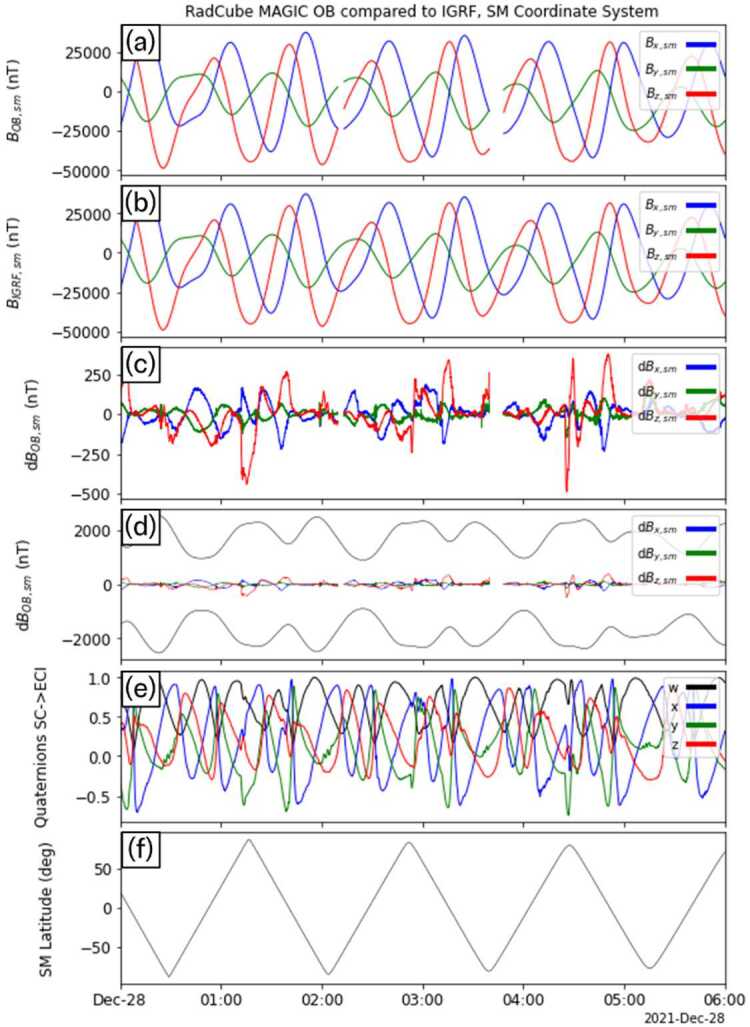
RadCube MAGIC data 2021-12-28 00:00 - 2021-12-28 06:00 in the SM coordinate system and compared to IGRF. Data are shown in the same format as Fig. 9

**Fig. 12:**
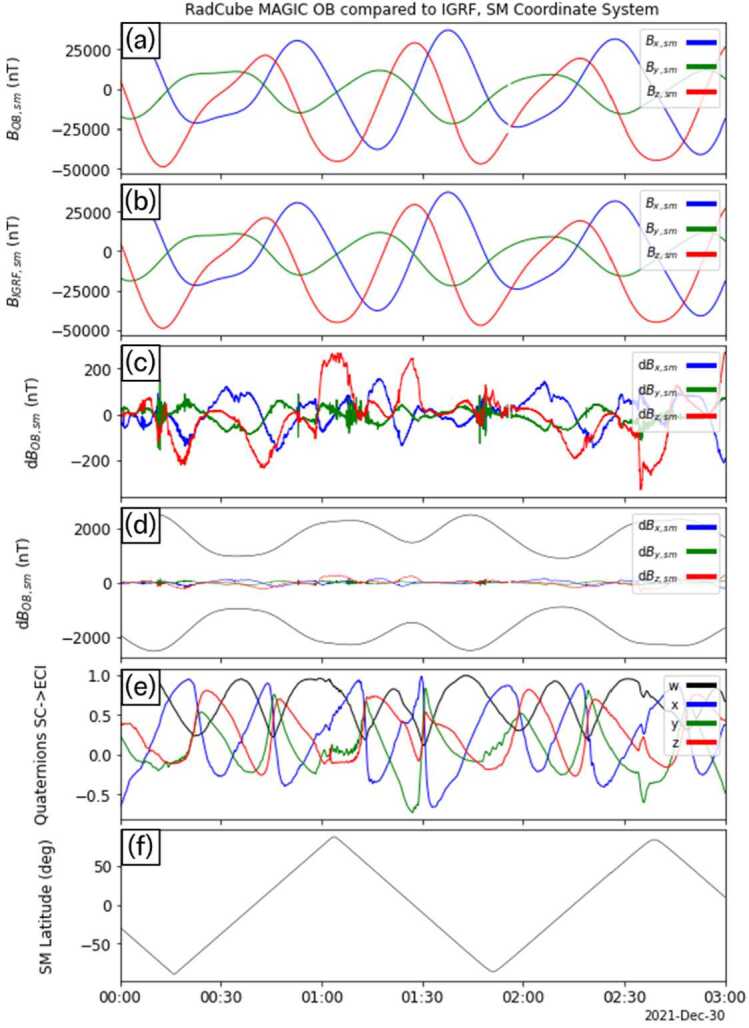
RadCube MAGIC data 2021-12-30 00:00 - 2021-12-30 06:00 in the SM coordinate system and compared to IGRF. Data are shown in the same format as Fig. 9

Figure 10, Fig. 11, and Fig. 12 show the results for the other three intervals of OB data in the same format as Fig. 9. In each interval, we find that the magnetic field is well measured by the OB sensor, and both the mission requirements and goals are met. It was therefore concluded that this represented a successful technology demonstration of the RadCube system and the MAGIC instrument.

### Boom Deployment and OB Sensor Noise Analysis

As introduced in Sect. 3, the boom deployment took place on 28 October 2021 during which time MAGIC was operational in FAST mode, with the OB sensor recording data at 10 vectors/s. Given the short duration and changes in the magnetic environment, the calibration techniques in Sect. 4.1 could not be applied. The data were therefore calibrated using the average OB parameters derived above and summarised in Table 6. The data were calibrated without reference to the temperature, as the temperature of the sensor just prior to deployment was measured to be approximately 6 °C. Once the OB sensor was moving away from the satellite and fully illuminated, the temperature increased to 23 °C by the end of the interval. This relatively rapid temperature change may also imply temperature gradients across the OB housing, and therefore that once deployed, the measured temperature may not accurately reflect the temperature of the sensor.

**Table 6:** Calibration parameters applied to the RadCube MAGIC boom deployment interval

Calibration Parameter		Value
x-axis angles [°]	$(\theta _{\mathrm{x}}, \phi _{\mathrm{x}})$	(88.82,0.00)
y-axis angles [°]	$(\theta _{\mathrm{y}}, \phi _{\mathrm{y}})$	(88.92,91.87)
z-axis angles [°]	$(\theta _{\mathrm{z}}, \phi _{\mathrm{z}})$	(0.01,0.00)
Offsets [nT]	$(\mathrm{O}_{\mathrm{x}}, \mathrm{O}_{\mathrm{y}}, \mathrm{O}_{\mathrm{z}})$	(340,650,20)
Gains	$(\mathrm{G}_{\mathrm{x}}, \mathrm{G}_{\mathrm{y}}, \mathrm{G}_{\mathrm{z}})$	(1.030,1.010,1.018)

**Fig. 13:**
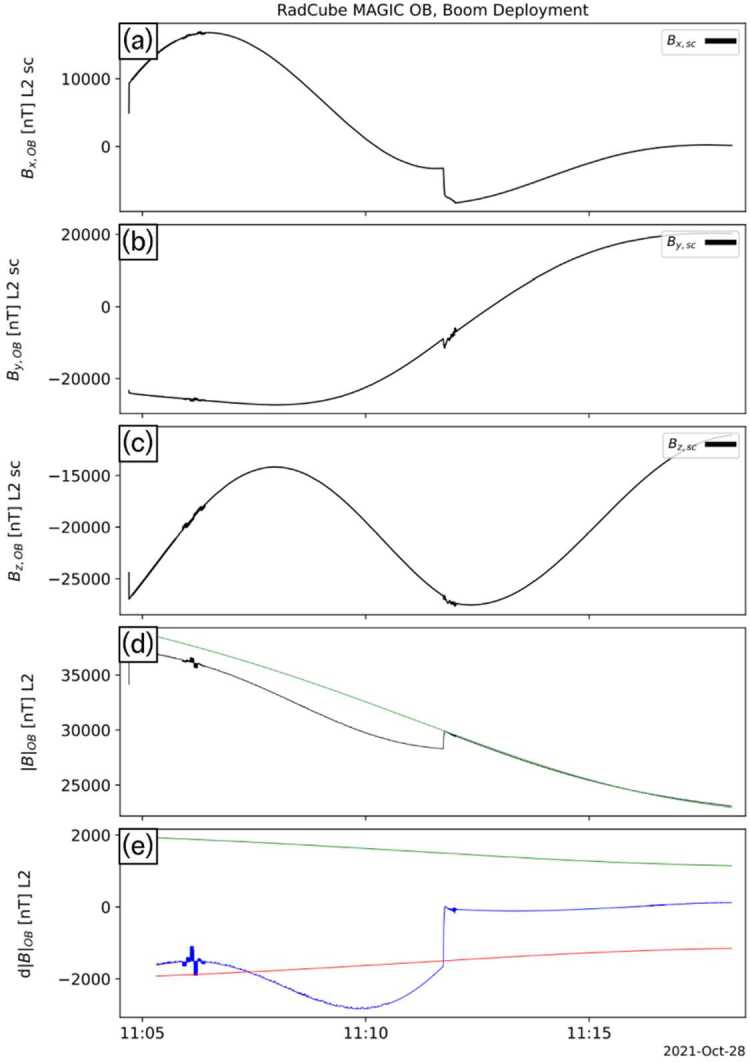
RadCube MAGIC observations during the boom deployment interval on 28 October 2021. Panels (a-c): x, y, and z components of the L2 calibrated magnetic field data measured by RadCube in the sensor coordinate system. (d) Measured magnetic field strength (black) and expected IGRF magnetic field strength (green). (e) Difference between the observed and model magnetic field strength (blue), with 5% error envelope (red/green). The boom deployment corresponds to the step change visible in panels (a), (d), and (e)

The calibrated magnetic field data acquired during the entire boom deployment interval are shown in Fig. 13. Panels (a-c) show the three components of the measured magnetic field in sensor coordinates. Panel (d) shows the measured magnetic field strength (black) compared to the expected IGRF magnetic field strength (green). Panel (e) shows $\text{d}|\text{B}|_{\text{OB}} = |\text{B}|_{\text{OB},\text{L2}} - |\text{B}|_{\mathrm{IGRF}}$, the difference between the measured and expected field strength (blue), and the 5% error envelope (red/green).

Before the boom deployment, the ADCS magnetotorquer system was fired generating a series of pulses that are visible at ∼11:06 UT. In addition, magnetic noise is also visible, for example in panel (e). The boom deployment corresponds to the apparent step change seen in panel (d) and (e). After deployment, the measured field closely agrees with the expected field from the IGRF model. A second magnetotorquer firing performed after the boom deployment is not evident on this plot, further confirming the successful deployment.

**Fig. 14:**
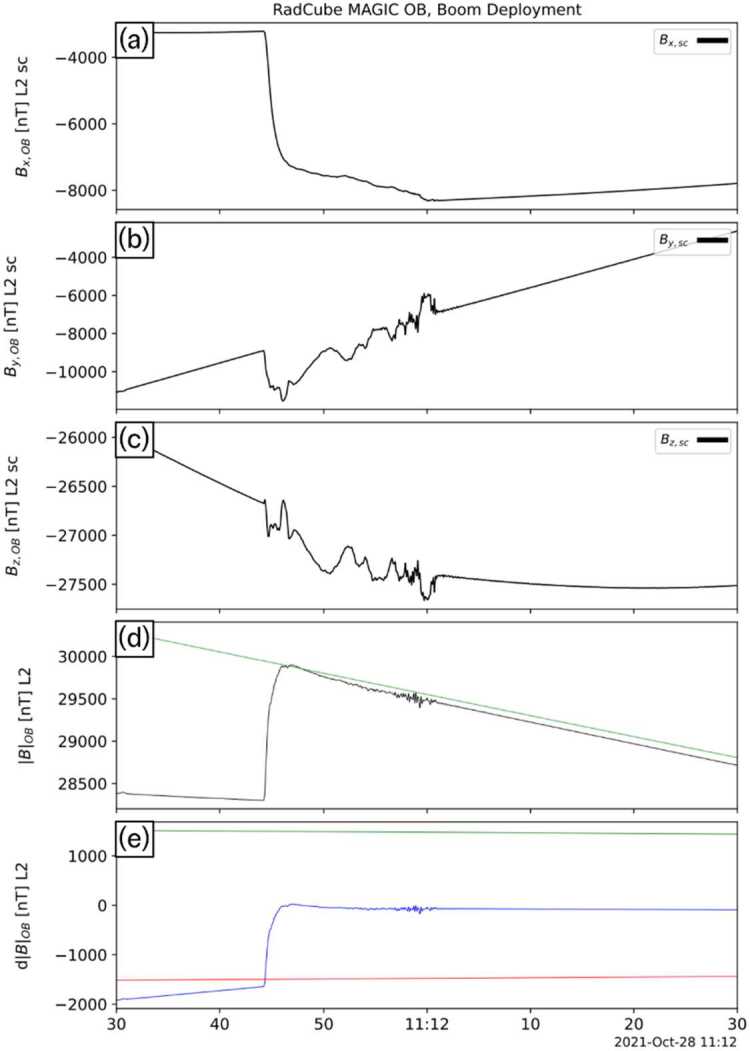
Detail of observations made during the RadCube boom deployment, presented in the same format as Fig. 13

Figure 14 shows the boom deployment interval in more detail. The deployment starts at 11:11:44 UT, with the boom deploying to a length of 80 cm during a period of approximately 15 s. Significant fluctuations were observed during the deployment, as expected due to the mechanical motion of the boom. These fluctuations do not persist after the boom deployment is complete. The close agreement of the post-deployment observations with the expected magnetic field strength, derived using calibration parameters from the subsequent observations, demonstrates the validity of the calibration solution.

**Fig. 15:**
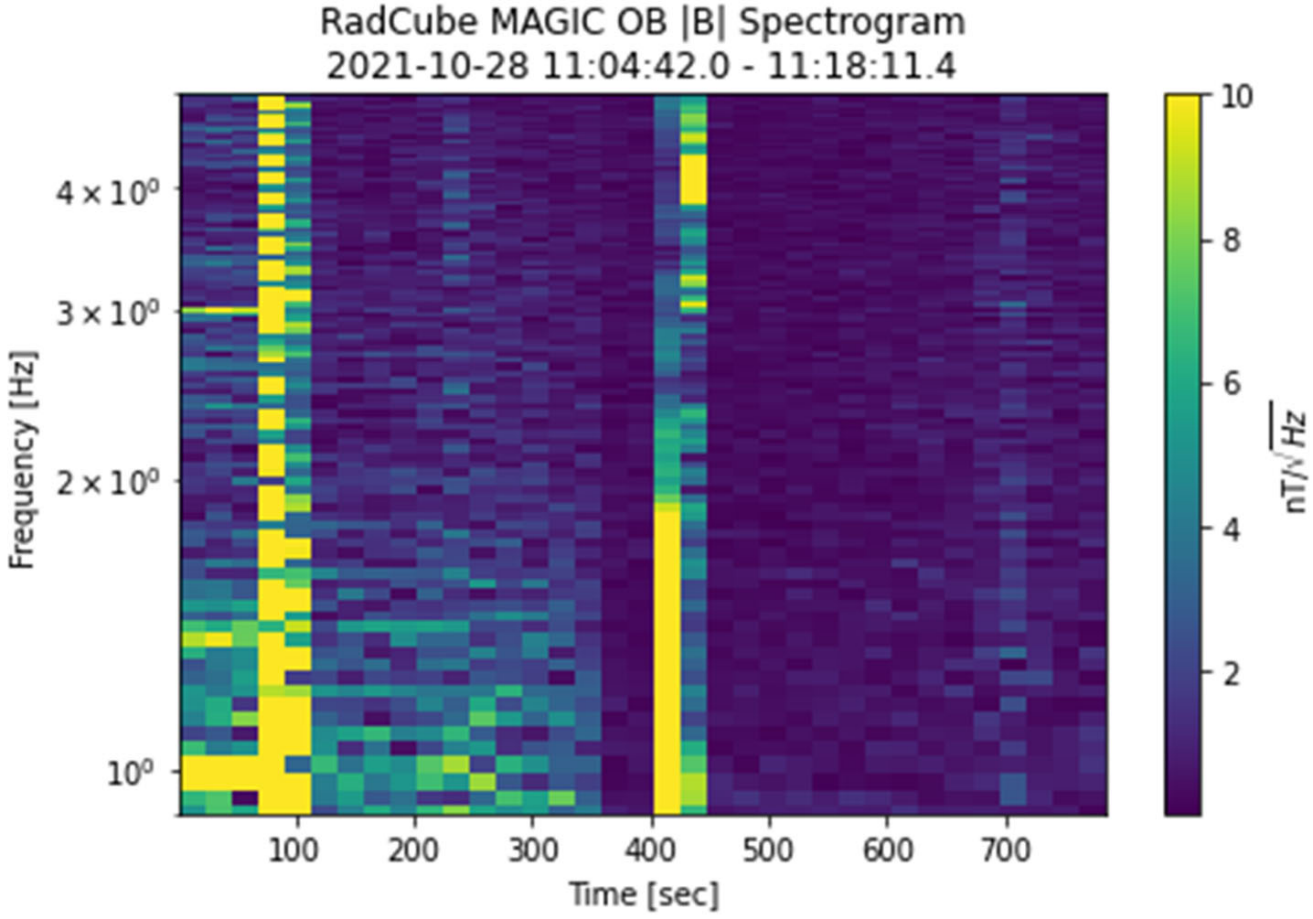
Spectrogram of the magnetic field strength, during the boom deployment interval. 11:04:39 UT corresponds to $t = 0$. The plot shows the power spectral density (PSD) in units of nT/$\sqrt{\text{Hz}}$

Figure 15 shows a spectrogram of the magnetic field strength measured throughout the entire boom deployment interval, corresponding to the time period 11:04:42 – 11:18:11.4 UT, revealing further information about the changes in the magnetic environment of the OB sensor. At the start of the interval, the sensor is undeployed, and as noted above the environment is relatively noisy. At $t \sim 100\text{ s}$, the ADCS magnetotorquer system was fired, introducing a short-lived broad-band signal in the spectrogram. There is a drop in noise just prior to the boom deployment interval (this occurred at $t = 422\text{ s}$, and is also visible in Fig. 13). Once the boom deployment is complete, the noise environment of the sensor is much quieter. A second magnetotorquer firing occurred at $t \sim 700\text{ s}$ and whilst detectable, its amplitude was substantially reduced. This further confirmed the successful boom deployment.

**Fig. 16:**
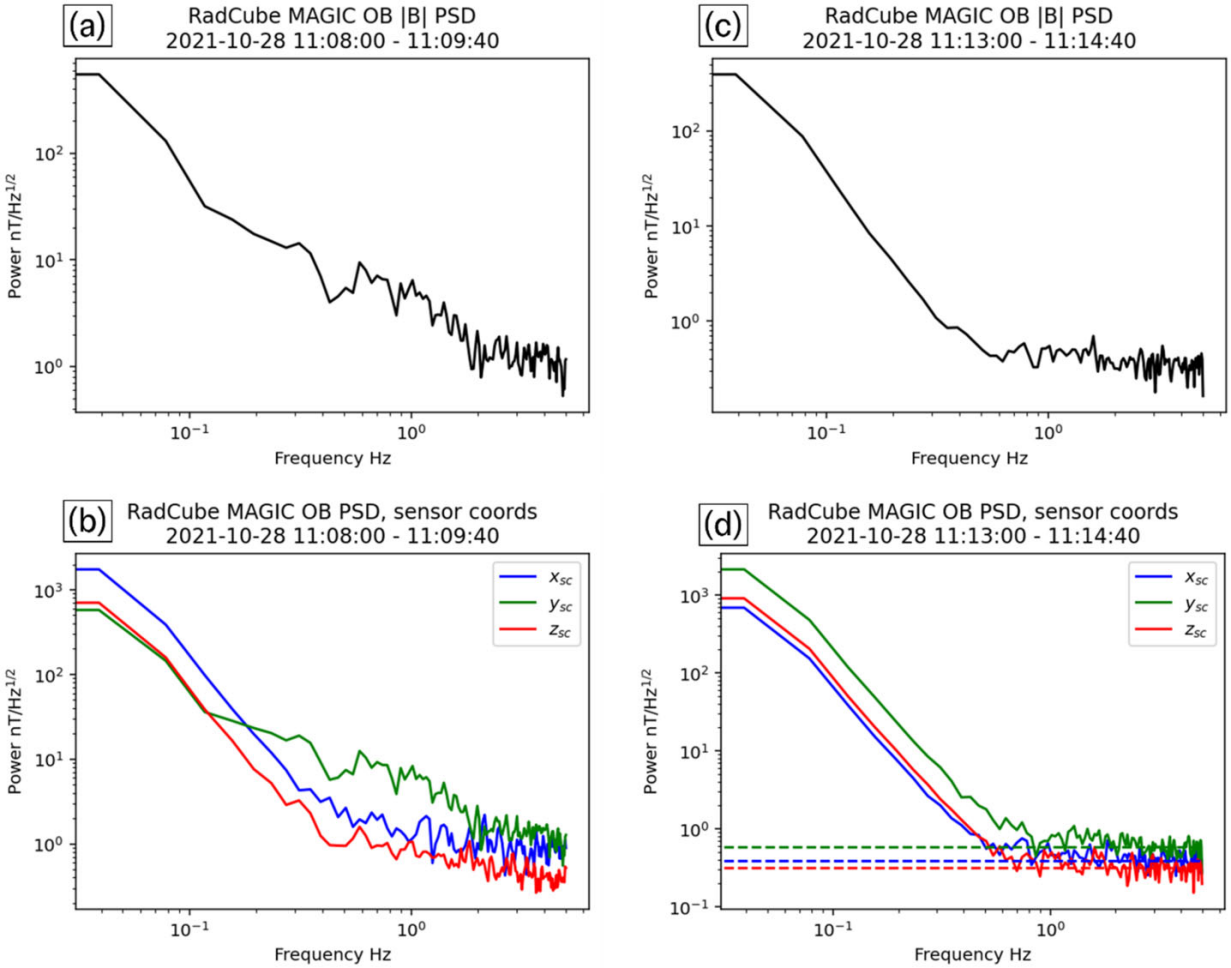
Noise performance of RadCube MAGIC before and after boom deployment. Panel (a) Amplitude Spectral Density (ASD) of |B| pre-deployment. Panel (b) ASD of the magnetic field components pre-deployment in the sensor coordinate system (x: blue, y: green, z: red). Panel (c) ASD of |B| post-deployment. Panel (d) ASD of the magnetic field components post-deployment in the sensor coordinate system (x: blue, y:green, z:red). Horizontal dashed lines show the estimated noise floor in each component

To further understand the performance of the OB sensor two intervals, pre-and post-deployment, were analysed and compared. The pre-deployment interval is 11:08:00 – 11:09:40 UT and the post-deployment interval is 11:13:00 – 11:14:40 UT. Amplitude Spectral Density (ASD) curves were computed for both the field strength and the components, and are summarised in Fig. 16. Panels (a-b) show the pre-deployment data and panels (c-d) show the post-deployment data, respectively. A reduction in the power in all curves is observed post-deployment. The estimated noise floor in the post-deployment interval is marked in Fig. 16(d), finding that $\text{P}_{\text{Bx}} = 368\text{ pT}/\sqrt{\text{Hz}}$, $\text{P}_{\text{By}} = 599\text{ pT}/\sqrt{\text{Hz}}$ and $\text{P}_{\text{Bz}} = 317\text{ pT}/\sqrt{\text{Hz}}$. As expected, the noise floor is somewhat elevated relative to laboratory testing, most likely due to the variation in the natural environment and residual magnetic noise from the platform. Nevertheless, a significant improvement in in-flight performance is found compared to the CINEMA observations, which exhibited an inflight noise level of $1.35\text{ nT}/\sqrt{\text{Hz}}$ (Archer et al. 2015).

### Discussion of the OB Sensor Performance

Whilst RadCube provided a successful technology demonstration of the MAGIC instrument, with measurements from the OB sensor surpassing the magnetic field measurement requirement and goal, a number of challenges were encountered in flight which we now discuss. An important aspect of technology demonstration missions is that by definition they accept higher risk in terms of degradation in performance and/or failure, since the technology has not been previously flown. Understanding deviations from expected behaviour is therefore an important opportunity to gain critical insight that may to inform future development and implementation.

Following the start of nominal operations in November 2021, a periodic anomaly was observed in the OB sensor performance, whereby the three-axis measurement saturated to a constant strength and orientation before recovering to normal operation. This was found to correlate with the temperature measured on the OB sensor, initially occurring when the OB sensor temperature exceeded a threshold of approximately 60 °C. It was concluded that this was consistent with heating of the sensor on the dayside by solar illumination.

Upon identifying this behaviour, significant effort was applied to replicating the fault on the ground, and establishing its root cause. The EQM model was tested and no saturation was observed even above 80 ^∘^C. Some evidence for anomalous behaviour was identified above the upper qualification temperature on the PFM during testing, but not in the temperature range seen in orbit. The fact that the inboard sensor was unaffected led to the conclusion that that this was not caused by an electrical fault in the set-reset pulse generation.

Subsequently, three possible hardware faults were identified: failure of the set-reset strap in one of the AMR chips, failure of the ESD sensitive hybrid Micrel 4424, and failure of an electrical path through thermal expansion within the harness, connector, or sensor. A new test sensor was manufactured, but it was not found possible to replicate the failure as a function of temperature. Furthermore, a full thermal-vacuum test campaign on the EQM sensor was successfully completed in April 2022 when facilities were once again available.

To mitigate this problem, it was decided to implement a campaign to shadow the OB sensor, by controlling the orientation of RadCube such that the sensor would always be located in the satellite’s shadow. This was completed successfully, also demonstrating the precision of the ADCS on RadCube, and RadCube was operating in this mode when acquiring the four extended intervals of OB data shown in Fig. 9 - Fig. 12.

As the mission progressed, a second recoverable failure began to be observed when the OB sensor became too cold. The threshold temperature for cold failure of the OB sensor was initially as low as −20 °C, but rose during Jan-Feb 2022 to the range 10 – 20 °C. A possible cause is generation of mechanical stress on harness by thermal cycling, leading to degradation and failure of an electrical connection. Beyond 8 Feb 2022, no OB data could be recovered.

Whilst there is no definitive analysis of the reason for the OB sensor failure, as it would appear impossible to replicate the necessary flight conditions on the ground, a plausible scenario is considered to be mechanical motion associated with the continuous temperature cycling in low Earth orbit. In this scenario, small changes in mechanical properties caused by thermal effects placed repeated periodic stress on the harness or the connection of the harness into the OB sensor. Other mechanical effects have been considered as well, for example frictional abrasion of the harness against the boom. Under such circumstances, this could cause degradation of the sensor performance through a variety of failure modes (e.g. broken contacts, damaged harness wires, EM interference across harness wires).

In a future implementation it will be necessary to examine more carefully the thermal environment in LEO, and undertake more detailed testing of the entire system to better understand these issues. On RadCube the Covid-19 pandemic significantly impeded pre-launch activities in the build phase, and for future missions development, it should be possible to undertake a considerably more comprehensive testing programme.

## Inboard (IB) Sensor Performance

The degradation of the OB sensor performance led to closer investigation of the IB sensor performance and the extent to which it was capable of also meeting the mission goals. When RadCube was three-axis stabilised, the IB data measured in NORMAL mode at 0.1 vectors/s was found to contain very significant satellite-generated signals and was not useable. However, the very low noise environment of the OB sensor meant that it was not necessary to attempt gradiometry or other OB/IB comparisons. As such the IB data from the first part of the mission was not further investigated.

However, although RadCube is designed to be three-axis stabilised, it can also fly in a so-called ‘ADCS-ready’ mode, where the platform orientation is uncontrolled and it slowly tumbles on timescales of minutes. During operations, RadCube would enter this mode for short periods of time, and it was noticed that the platform was extremely magnetically quiet when operating in this mode. Following further tests, a new mode of operation was designed and implemented from 4 Apr 2022 with the IB sensor being set as the primary sensor in NORMAL mode measuring at 1 vector/s, and RadCube slowly tumbling in ADCS-ready mode for an extended period of time. During the ADCS-ready mode operations no OB data was available as this took place after the failure of the OB sensor.

In Sect. 5.1 we present a case study of IB primary sensor data from 29 – 30 Apr 2022 that demonstrates the successful application of the attitude independent calibration procedure (described in Sect. 4.1). 17 intervals of data similarly acquired between 4 April 2022 and 1 May 2022 (the end of the main RadCube mission) were then analysed. Reviewing the average calibration parameters of the IB sensor over this period, we find that the IB sensor performance also meets the RadCube mission requirement and goal.

In the ADCS-ready mode, the control system does not provide a quaternion solution, but perhaps surprisingly, the slow tumbling of RadCube does not invalidate the assumptions of the quaternion reconstruction procedure. In Sect. 5.2 we demonstrate successful reconstruction of the quaternions for the case study interval in Sect. 5.1. Moreover, we find that the absence of short-lived platform attitude changes (as seen during the OB data acquisition when RadCube is in a three-axis stabilised mode) means that there are no corresponding variations in the observed magnetic field components in the sensor frame on time scales of 10-100 s. This means that deviations from IGRF are clearly observed in the data and can be identified as Field Aligned Current (FAC) signatures. An analysis of signatures consistent with FACs in the 29-30 April 2022 case study event is presented in Sect. 5.3.

The RadCube extended mission continued until the end of 2022, and a further health check of the MAGIC instrument was performed in November 2023, more than two years after launch. We briefly review the health check data in Sect. 5.4, finding no further degradation in the performance of MAGIC. Finally, in Sect. 5.5 we discuss the overall performance of the IB sensor and implications for future missions.

### Attitude Independent Calibration of the Inboard (IB) Sensor

**Fig. 17:**
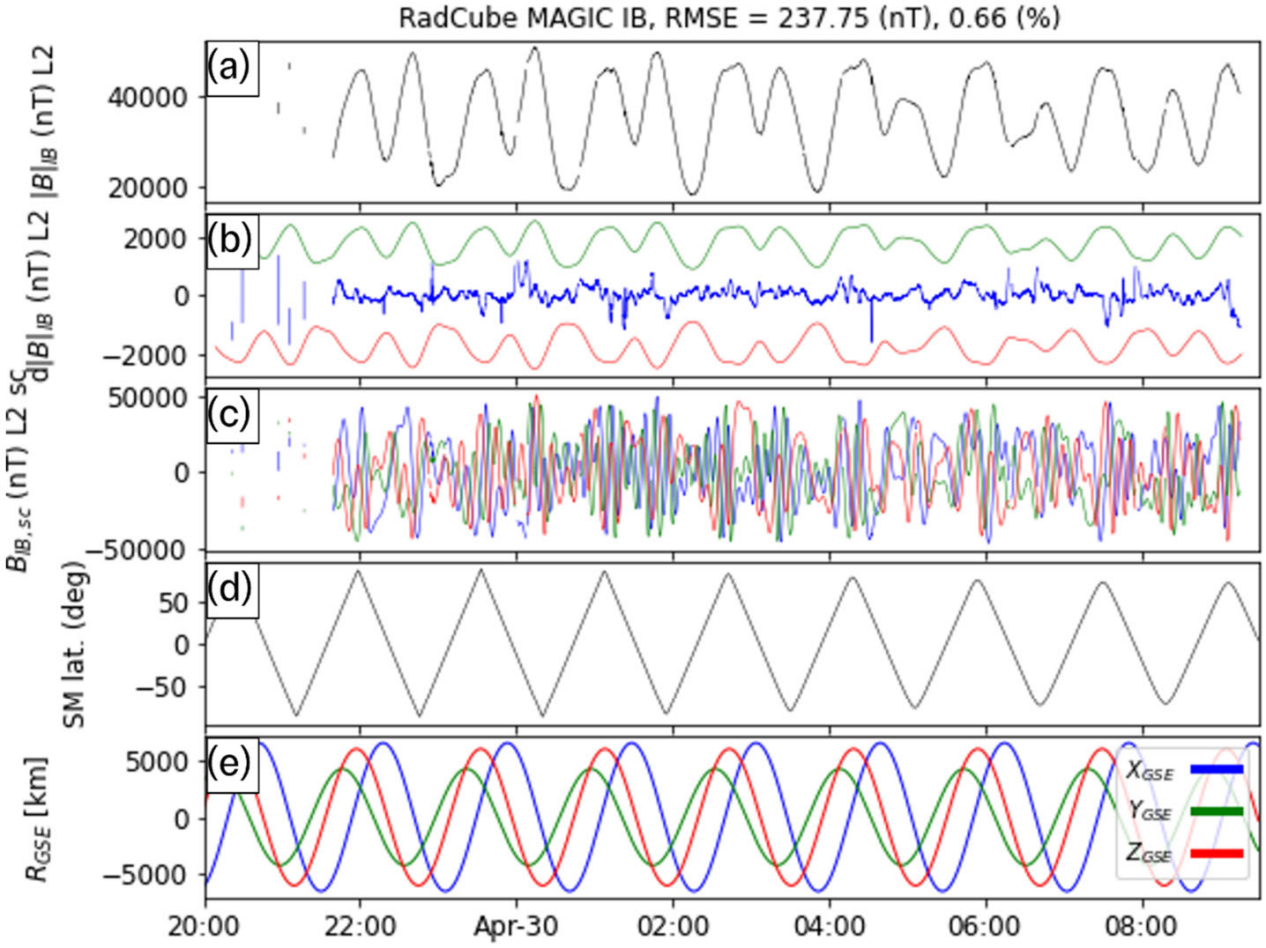
Calibrated IB data from 2022-04-29 20:00 - 2022-04-30 09:00. Panel (a): L2 calibrated magnetic field strength measured by RadCube. (b) Difference between the observed and model magnetic field strength (blue), with 5% error envelope (red/green). (c) L2 calibrated magnetic field in the sensor coordinate system (x: blue, y:green, z: red). (d) Solar Magnetic latitude of RadCube. (e) Location of RadCube in Geomagnetic Solar Ecliptic coordinates (x: blue, y:green, z:red)

An example of IB sensor performance is presented in Fig. 17, corresponding to observations made on 2022-04-29 20:00 - 2022-04-30 09:00. This interval corresponds to a period of enhanced geomagnetic activity with a Kp index of 3. The data are presented in the same format as the OB data in Fig. 9, and have been calibrated using the attitude independent procedure described in Sect. 4.1. In this example the RMSE of the magnetic field strength is 237.75 nT, and the fractional RMSE is 0.66%.

To better understand the performance of the IB sensor, 17 intervals of data acquired up to the end of the main mission were similarly analysed and the average calibration parameters are summarised in Table 7. The angle corrections and gains are found to be relatively stable, as for the OB sensor. The variation in the offsets are more significant from event to event. This is thought to reflect the changing platform magnetic environment for each measurement interval and could also be due to offset drift with time. Overall, the average RMSE is found to be ∼320 nT, with a fractional RMSE error of 0.9%. This demonstrates that the performance of the platform-mounted IB sensor also meets the mission goal, when RadCube was operating in the ADCS-ready mode that minimises the platform magnetic interference. The performance is also superior to the CINEMA inboard sensor, where a fractional RMSE error of 1.95% was reported (Archer et al. 2015).

**Table 7:** Average calibration parameters for 17 intervals of Inboard sensor data acquired between 4 April 2022 and 1 May 2022

Calibration Parameter	Average	Std
Time Offset [s]	−6.2	95.8
*θ*_x_ [°]	85.985	1.036
*ϕ*_x_ [°]	0.012	0.004
*θ*_y_ [°]	89.221	0.393
*ϕ*_y_ [°]	89.11	0.446
*θ*_z_ [°]	0.016	0.003
*ϕ*_z_ [°]	−0.003	0.004
L2 O_1,x_ [nT/°C]	4.33	8.97
L2 O_1,y_ [nT/°C]	−1.46	10.97
L2 O_1,z_ [nT/°C]	4.27	11.85
L2 O_2,x_ [nT]	1000.73	1168.38
L2 O_2,y_ [nT]	−1332.6	956.18
L2 O_2,z_ [nT]	1797.48	2986.53
L2 G_x_	0.989	0.01
L2 G_y_	0.966	0.028
L2 G_z_	1.042	0.024
L2 RMSE [nT]	324.839	113.148
L2 Fraction	0.009	0.003

### Quaternion Reconstruction

In this section we present a case study applying the quaternion reconstruction technique to IB data. We reconstruct the quaternions over a 30-minute period, 2022-04-30 02:30 – 03:00 UT, during the interval shown in Fig. 17. Following the same procedure, the quaternions were successfully reconstructed. However, because RadCube was undergoing slow tumbling, the data used for the reconstruction were low-pass filtered with a cutoff of 0.01 Hz, a lower frequency than used for the OB data analysis, to avoid contamination with any potential geophysical signatures. The quaternions were then used to rotate the magnetic field data from sensor coordinates into Solar Magnetic coordinates.

**Fig. 18:**
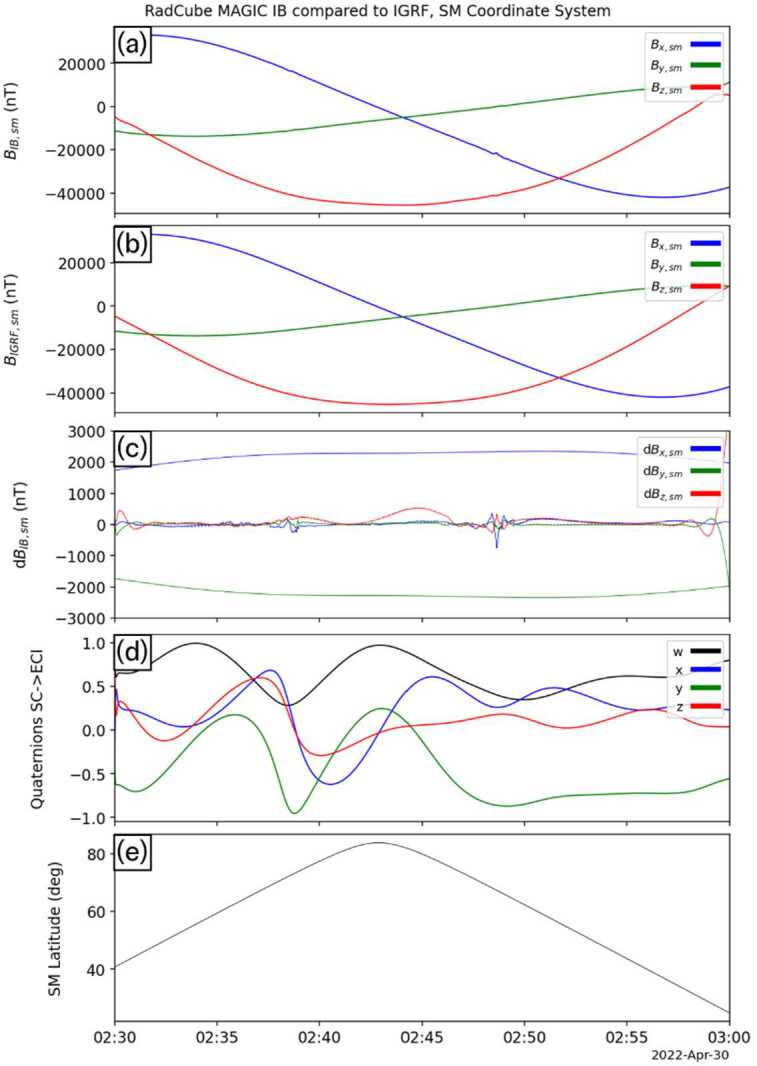
Calibrated IB data from 2022-04-30 02:30 - 2022-04-30 03:00 in the SM coordinate system and compared to IGRF. Panel (a): L2 calibrated magnetic field data measured by RadCube in the SM coordinate system (x: blue, y: green, z: red). (b) IGRF magnetic field expected at the location of RadCube in SM coordinates (x: blue, y: green, z: red). (c) Difference between each component of the observed and model magnetic field (x: blue, y: green, z: red), also showing the 5% error envelope based on the magnetic field strength. (d) Reconstructed quaternions (w: black, x: blue, y, green, z: red). (e) Latitude of RadCube in SM coordinates

The data are presented in Fig. 18 in the same format as the OB data shown in e.g. Fig. 9. Panels (a-b) show the calibrated IB magnetic field data in SM coordinates and the corresponding IGRF magnetic field data. There is very good agreement, confirmed by panel (c), which shows the difference in each component. as well as the 5% error envelope. Panel (d) shows the reconstructed quaternions, and panel (e) shows the SM latitude of the observation. Generally there is very close agreement (note that the larger deviation at the end of the interval is an artefact associated with the filtering process), and this demonstrates the capability of the IB sensor to also meet the RadCube mission goals, being able to measure the magnetic field in LEO to an accuracy of better than 1%.

Figure 18 also reveals two features where there are structured deviations between the observations and the model. Unlike the case of the OB sensor, these do not correspond to times when RadCube was rapidly changing orientation, as the reconstructed quaternions (panel d) remain smoothly varying throughout. These features occur just before and after the point of maximum SM latitude (panel e) and are analysed in more detail in the next section.

### Detection of Field Aligned Current Signatures

**Fig. 19:**
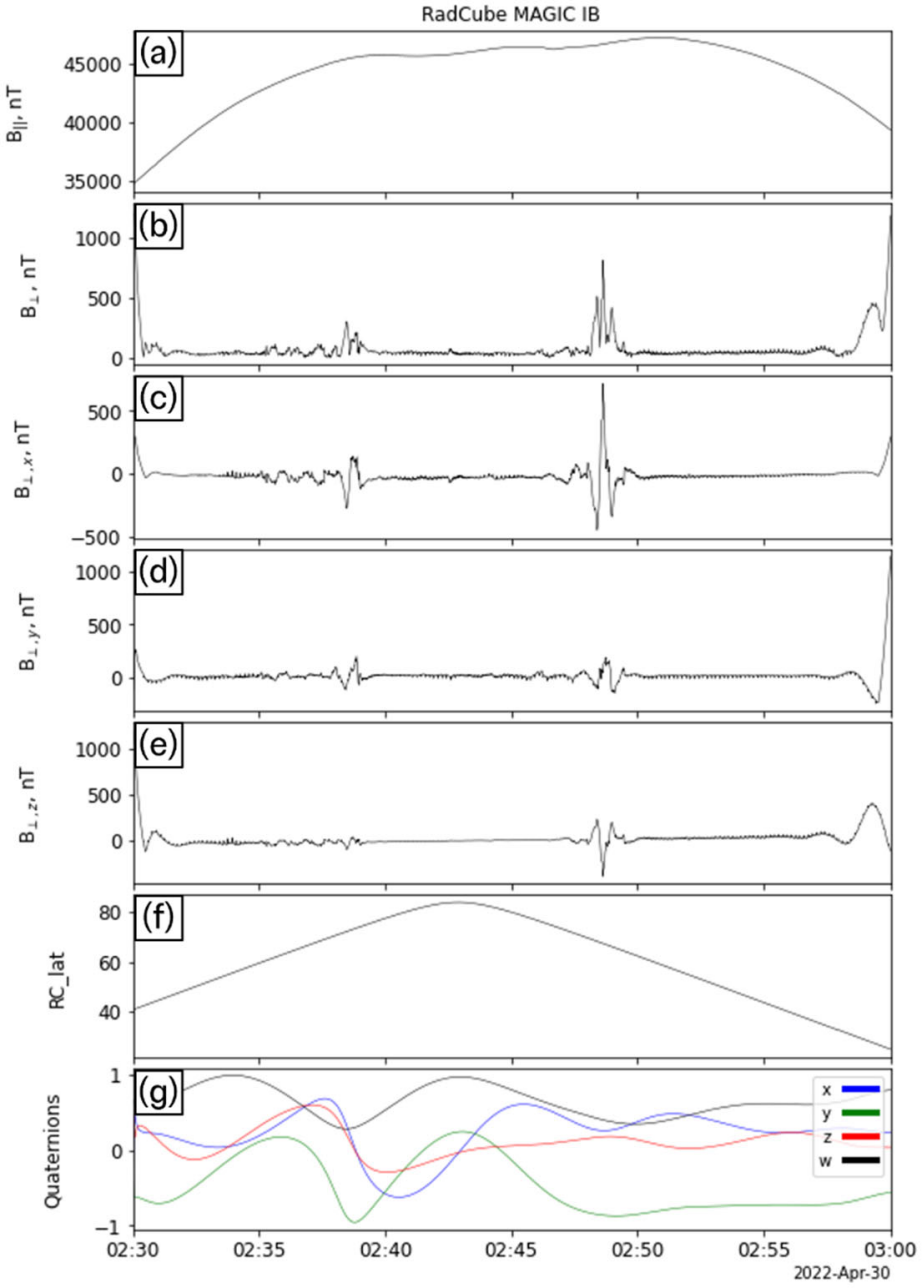
RadCube IB MAGIC data 2022-04-30 02:30 - 2022-04-30 03:00 in field aligned coordinates. Panel (a-b): Parallel and perpendicular components of the magnetic field. (b) Perpendicular magnetic field component (c-e) components of the perpendicular magnetic field in the SM coordinate system. (e) Latitude of RadCube in SM coordinates. (f) Reconstructed quaternions (w: black, x: blue, y, green, z: red)

To better understand differences between the observed and modelled field shown in Fig. 18, the data were rotated into a field aligned coordinate system. This coordinate system was reconstructed by first convolving the observed magnetic field data with 60 s square wave kernel, and then computing $\hat{\boldsymbol{b}}_{\mathrm{smth}}$, the orientation of smoothed magnetic field unit vector in SM coordinates. The parallel and perpendicular field components are then defined as $\boldsymbol{B}_{\mid \mid} =( \boldsymbol{B} \cdot \hat{\boldsymbol{b}}_{\mathrm{smth}} ) \hat{\boldsymbol{b}}_{\mathrm{smth}}$ and $\boldsymbol{B}_{\bot} = \boldsymbol{B} - \boldsymbol{B}_{\mid \mid} $, respectively. The results of this analysis are shown in Fig. 19. Panels (a-b) show the parallel and perpendicular field components, respectively. Panels (c-e) show the components of perpendicular field in SM coordinates. For context, panels (f-g) show the SM latitude of RadCube and the quaternions. The perpendicular field signatures occur just before and after the time of maximum positive SM latitude.

Field aligned currents are a fundamental feature of magnetosphere ionosphere coupling (Cowley 2000; Milan et al. 2017). Since their first observation (Iijima and Potemra 1976, 1978), FACs in LEO have been extensively studied with a wide variety of space missions (Anderson et al. 2005; Dunlop et al. 2015; Forsyth et al. 2018; Gjerloev et al. 2011; Knipp et al. 2014; Potemra 1985; Weimer 1999). To further explore the RadCube observations, the field-aligned current density $j_{\mid \mid} $ is calculated assuming an infinite 1-d current sheet (Luhr et al. 1996) where

12 $$ j_{\mid \mid} = \frac{1}{\mu _{0} v_{sc,\bot}} \frac{d}{dt} \left ( \boldsymbol{B}_{\bot} \cdot \hat{\boldsymbol{n}} \right ). $$

Here

13 $$ \hat{\boldsymbol{n}} = \frac{\hat{\boldsymbol{b}}_{\mathrm{smth}} \times \boldsymbol{v}_{sc}}{\left \vert \hat{\boldsymbol{b}}_{\mathrm{smth}} \times \boldsymbol{v}_{sc} \right \vert} $$

is the unit vector perpendicular to both $\hat{\boldsymbol{b}}_{\mathrm{smth}}$ and $\boldsymbol{v}_{sc}$ (see Archer et al. 2015), $\boldsymbol{v}_{sc}$ is the spacecraft velocity in SM coordinates, and $v_{sc,\bot} $ is the spacecraft speed perpendicular to the magnetic field. Multi-point observations from the SWARM mission demonstrate that assumptions of stationarity apply best to large-scale FAC structures (McGranaghan et al. 2017), for example greater than 150 km in size, which here corresponds to a time period of 20 s (Lühr et al. 2015).

**Fig. 20:**
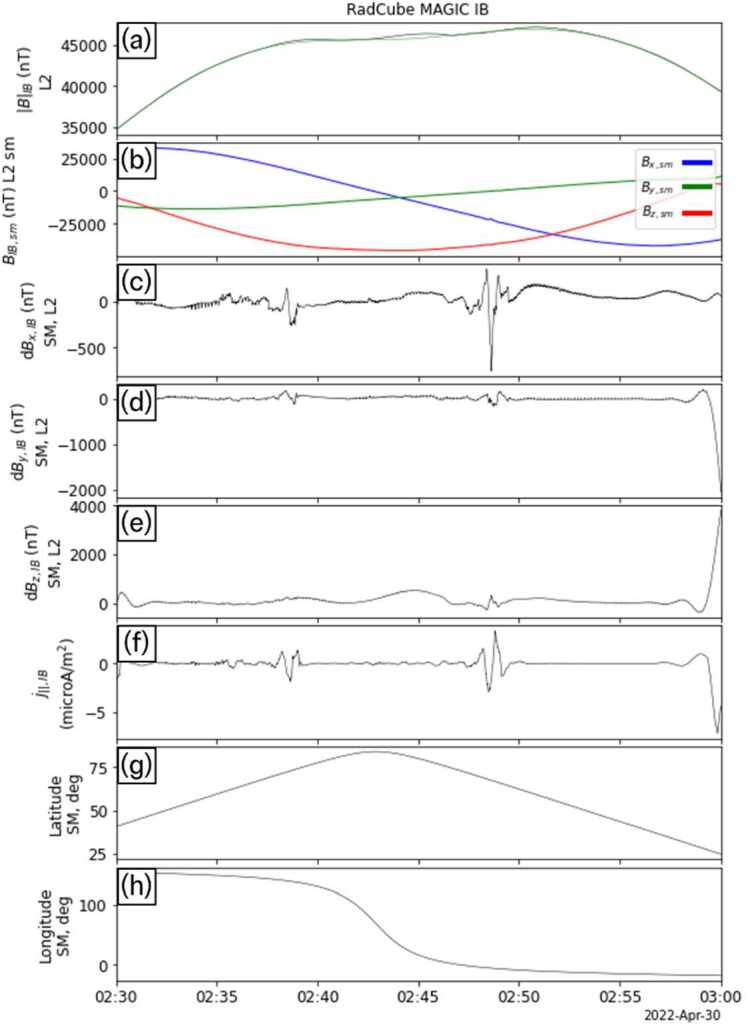
RadCube IB MAGIC data 2022-04-30 02:30 - 2022-04-30 03:00. Panel (a): Magnetic field strength observed by RadCube (black) and IGRF model (green). (b) Observed magnetic field components in SM coordinates. Panel (c-e) components of the perpendicular magnetic field in the SM coordinate system. Panel (f) Field aligned current density. Panel (g-h) Latitude and longitude of RadCube in SM coordinates

We therefore smooth the resulting $j_{\mid \mid} $ with a 20 s kernel to reveal more clearly the underlying large-scale structure. The results of this analysis are shown in Fig. 20. Panel (a) shows the observed magnetic field strength in black and the model magnetic field from IGRF in green. Panel (b) shows the field components in SM coordinates for context. Panels (c-e) show the difference between the observed and model field for each component in SM coordinates, and panel (f) shows $j_{\mid \mid} $. Finally, panels (f-g) show the SM latitude and longitude of RadCube. Two intervals of $j_{\mid \mid} $ were observed, first on the nightside (latitude increasing) at 02:38 – 02:40 UT, and the second on the dayside (latitude decreasing) at 02:48 – 02:50.

**Fig. 21:**
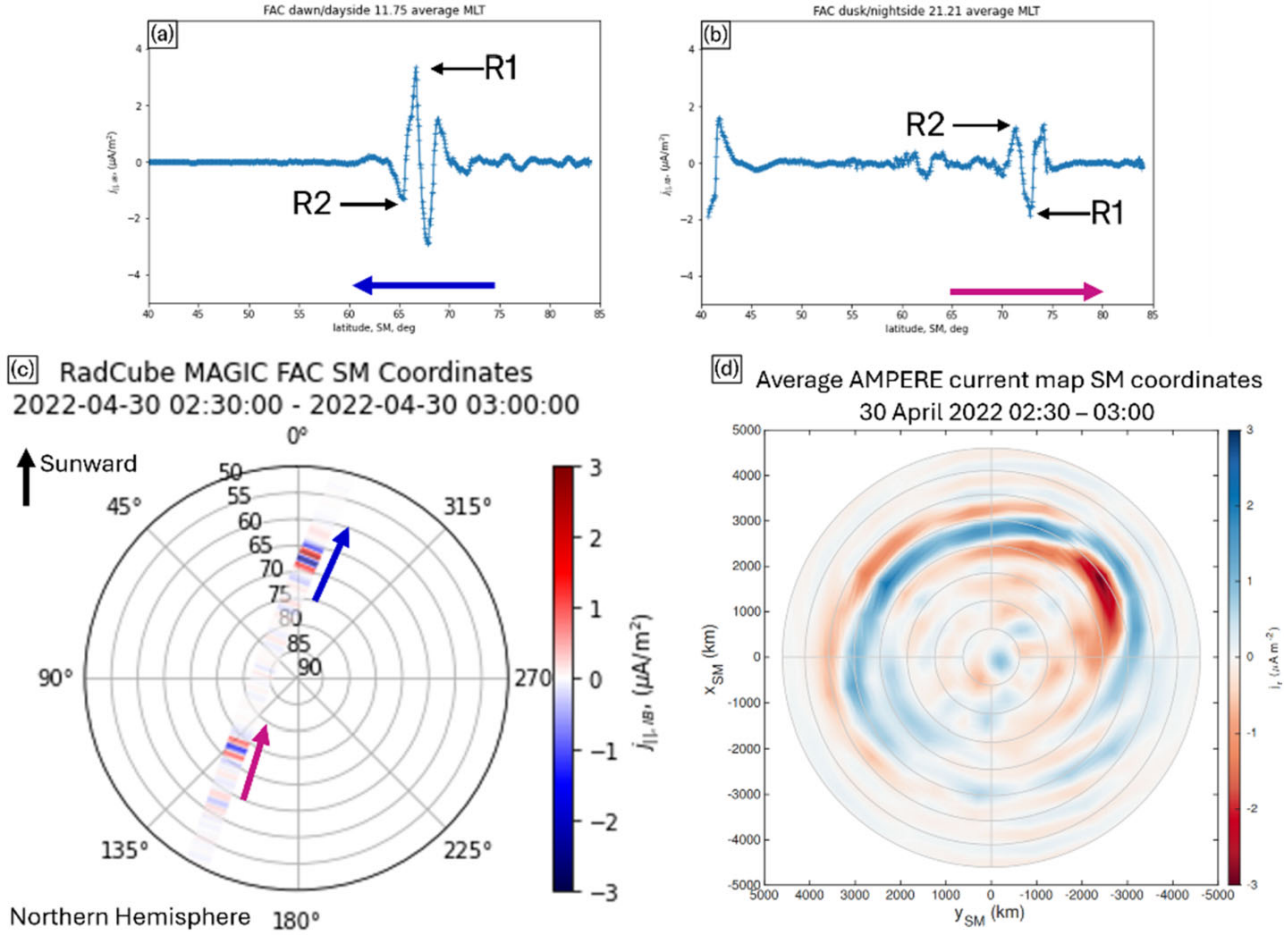
RadCube MAGIC field aligned current signatures observed on 2022-04-30, with AMPERE radial current map for context. Panel (a) ‘Dayside’ segment of field aligned current observations as a function of SM latitude observed between 02:42:54 – 03:00:00. Panel (b) ‘Nightside’ segment of field aligned current observations as a function of SM latitude observed between 02:30:00 – 02:42:54. Panel (c) field aligned current along the RadCube orbit track in SM coordinates. Panel (d) Average AMPERE radial current map in SM coordinates over the interval 02:30 – 03:00

The time of maximum SM latitude was 02:42:54 UT, and to further analyse the observations, the data were split into ‘nightside’ (when RadCube was ascending in SM latitude) and ‘dayside’ (when RadCube was descending in SM latitude) segments. The field aligned current observed in these segments is plotted as a function of SM latitude in Fig. 21(a-b). On the dayside, observed between 02:42:54 – 03:00:00 and shown in panel (a), the field aligned currents occurred between 65° – 70° SM latitude, at an average Local Time of 11.75. On the nightside, observed between 02:30:00 – 02:42:54, and shown in panel (b), field aligned currents were found to occur between 70° – 75° SM latitude, at an average Local Time of 21.21. The nightside field aligned currents were found to be weaker than on the dayside, and on the dayside, peak field aligned currents exceeding 2 $\mu $Am^−2^ were observed.

Figure 21(c) shows the field aligned current plotted along the orbit track of RadCube in SM coordinates. RadCube was moving from the nightside pre-midnight sector to the dayside pre-noon sector over the north pole, indicated by the red and blue arrows. For this trajectory, on the dayside we would expect to first observe a field-aligned region 1 current together with an anti-field aligned region 2 current at lower latitude (Cowley 2000). Tentative identification of current structures consistent with this pattern is shown in Fig. 21a, where the first positive peak in $j_{\mid \mid} $, which is also the largest signature, is identified as a region 1 current. On the nightside we would expect RadCube to first observe a field aligned region 2 current followed by an anti-field aligned region 1 current at higher latitude. Tentative identification of current spikes that are qualitatively consistent are shown in Fig. 21b.

To place the RadCube observations in context, Fig. 21(d) shows an AMPERE (Active Magnetosphere and Planetary Electrodynamics Response Experiment) map of the radial current density derived from engineering magnetometer data provided by the Iridium NEXT constellation (Anderson et al. 2021, 2014, 2002, 2000; Waters et al. 2020, 2001). The map is averaged over 02:30 – 03:00 UT since the spacing of satellites within orbital planes imposes a maximum time resolution of 10 minutes for independent time steps (Waters et al. 2020). The AMPERE data thus provides the expected location of regions of FACs during the RadCube observation period. We find that the latitude ranges at which RadCube observed FAC signatures are broadly consistent with AMPERE on both the day- and nightside.

However, detailed comparisons of the FAC structures observed by RadCube and AMPERE must be approached with care. As described above, the RadCube data analysis procedure requires first applying a 0.01 Hz low-pass filter, and so any magnetic structures in the data observed on timescales greater than 100 s will not be identified as FAC signatures. A timescale of 100 s corresponds to roughly 7° of latitude, and so here RadCube data can only be used to identify FAC structures at scales smaller than this. In contrast, the nature of the AMPERE data and the fitting procedure imposes a minimum latitudinal scale of approximately 7° (Waters et al. 2020). RadCube thus reveals structured FAC regions and multiple upward and downward current layers on smaller scales located within the broader region of FACs identified by AMPERE. Such structuring is likely to correspond to multiple arc structures as reported in other FAC observations (Wu et al. 2017) and demonstrates how in this example AMPERE and RadCube are complementary.

### November 2023 Health Check Performance

**Fig. 22:**
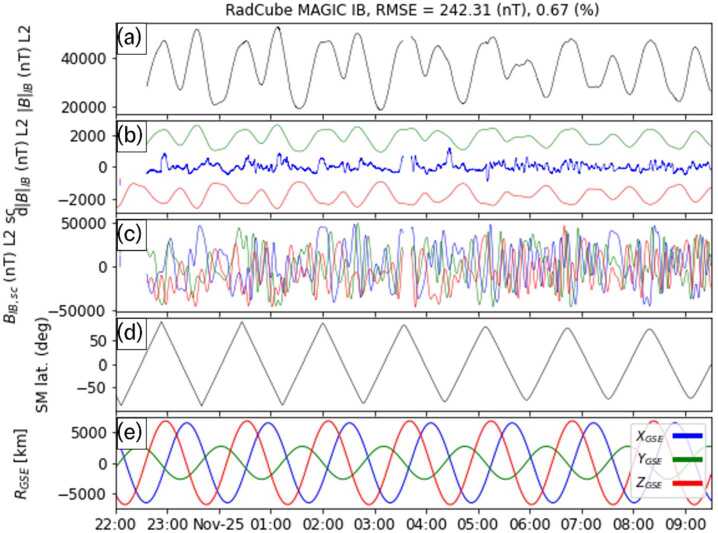
RadCube IB data from 2023 health check, acquired on 2023-11-24 22:00 - 2023-11-25 09:30. Panel (a): L2 calibrated magnetic field strength measured by RadCube. (b) Difference between the observed and model magnetic field strength (blue), with 5% error envelope (red/green). (c) L2 calibrated magnetic field in the sensor coordinate system (x: blue, y: green, z: red). (d) Solar Magnetic latitude of RadCube. (e) Location of Radcube in Geomagnetic Solar Ecliptic coordinates (x: blue, y: green, z: red)

In November 2023, a ‘health check’ of the RadCube MAGIC instrument was performed to examine its long-term performance more than two years after lauch. The MAGIC instrument was switched on for several periods of observation, with the IB sensor as primary and RadCube in ADCS-ready mode as before. An example interval of IB data from 24-25 November 2023 is shown in Fig. 22, presented in the same format as Fig. 17. The attitude independent calibration returned a RMSE of 241.99 nT, and a fractional RMSE of 0.67%. The calibration angles and gains were found to be stable compared to the 2022 observations, and no further reduction in the performance of the instrument was observed. The health check tests thus demonstrated the continuing functioning of the IB sensor and RadCube MAGIC after more than two years in orbit, with comparable performance to earlier measurements.

### Discussion of the IB Sensor Performance

Although the IB sensor was designed so that it could serve as the primary sensor (as described in Sect. 2), the discovery that RadCube platform was extremely magnetically quiet in ADCS ready mode was unexpected. The subsequent experiment to use the IB sensor as primary whilst RadCube operated in ADCS ready mode was designed and conducted in flight. This recovered a level of performance comparable to the OB sensor, and in fact enabled the detection of FAC signatures. As a result, the operating lifetime of the mission was also significantly extended.

This experiment also suggests that for magnetometry on small satellite platforms, it may be worth investigating the use of uncontrolled platforms that are extremely magnetically quiet, as this could lead to an engineering trade off where the sensor does not need to be placed at the end of a boom, further reducing the required resource envelope. However, this concept of operations is likely to be incompatible with other instrumentation on the same platform. Further work is required to understand how this could lead to an even more miniaturised magnetic field sensing platform that for space weather might operate as part of a wider network of satellites.

## Summary

As emphasised in the introduction, the measurement in space of magnetic fields is necessary for a wide variety of space science applications. With the rise of smaller satellite platforms and applications requiring a multi-point/constellation based approach, there is continued interest in the development of ever-more miniaturised technology. RadCube was implemented as a technology demonstration project to address these goals, and as part of the RadMag payload it carried the MAGIC instrument to measure the magnetic field in Low Earth Orbit. With a mass and power of 61 g and 0.48 W, the MAGIC resource envelope is an order of magnitude less than more traditional instrument solutions based on fluxgate technology. Within this envelope, MAGIC offers a dual sensor design and instrument intelligence as well as other capabilities described in Sect. 2.

Here we have reviewed the performance of MAGIC across more than two years in orbit. In general the instrument performance was superior to the previous iteration flown on the CINEMA CubeSat. Analysis of data from the OB boom-mounted sensor demonstrated performance exceeding both the mission and goal requirement. We therefore find that MAGIC demonstrated the ability to meet data product specifications for ESA space situational awareness monitoring. Further work will examine the performance of the OB sensor in more detail, including the measurements made during the boom deployment to better understand the RadCube magnetic environment. Finally, whilst the ultimate cause of the OB sensor failure is not precisely known, further detailed examination of the degradation will provide useful insight into how future versions of the instrument can be made more resilient to the large, repeated temperature changes that are experienced in LEO.

When set as primary, the IB sensor also demonstrated performance that exceeded the mission requirement and goal. The successful experiment to make measurements while RadCube was tumbling and magnetically quiet enabled regular operations, during which time magnetic field signatures consistent with Field Aligned Currents were observed. Based on the analysis presented here, the detectivity of FACs is comparable to CINEMA. However, CINEMA studies were frustrated by the very limited data return. Further work is required to fully survey the RadCube MAGIC dataset, as this is expected to recover further similar observations and enable exploration of instrument performance under a variety of geomagnetic conditions. In particular, the work presented here demonstrates that the MAGIC instrument may be used to characterise the properties of FAC signatures in LEO, which is an important space weather monitoring objective.

In conclusion, the flight of the MAGIC instrument on RadCube has enabled substantial progress in demonstrating the utility of anisotropic magnetoresistive sensors for monitoring the magnetic field in low Earth orbit. This extremely low-resource instrument solution is a natural complement to high-performance magnetometers carried on dedicated satellites. In the field of space weather, the use of MAGIC and other low resource instruments will further enable the development of envisaged future space weather monitoring systems requiring multi-point in situ observations from miniaturised platforms.

## Data Availability

The RadCube MAGIC magnetic field dataset presented here is publicly available and can be accessed via https://dx.doi.org/10.14469/hpc/14612. The MAGIC data analysis was performed in Python and made use of the PySPEDAS package (Grimes et al. 2022). AMPERE-NEXT data was obtained from https://ampere.jhuapl.edu/.
